# Divergent Trends in Insect Disturbance Across Europe's Temperate and Boreal Forests

**DOI:** 10.1111/gcb.70580

**Published:** 2025-10-29

**Authors:** Tomáš Hlásny, Roman Modlinger, Jostein Gohli, Rupert Seidl, Paal Krokene, Iris Bernardinelli, Simon Blaser, Gediminas Brazaitis, Gailenė Brazaitytė, Eckehard G. Brockerhoff, György Csóka, Laura Dobor, Maarten de Groot, Mihai‐Leonard Duduman, Massimo Faccoli, Margarita Georgieva, Georgi Georgiev, Wojciech Grodzki, Henrik Hartmann, Anikó Hirka, Gernot Hoch, Tomasz Jabłoński, Hervé Jactel, Mats Jonsell, Marija Kolšek, Markus Melin, Slobodan Milanović, Constantin Nețoiu, Mats Nieberg, Bjørn Økland, Milan Pernek, Michaela Perunová, Nick Schafstall, Martin Schroeder, Gottfried Steyrer, Jozef Vakula, Thomas Wohlgemuth, Tiina Ylioja, Andrew M. Liebhold

**Affiliations:** ^1^ Faculty of Forestry and Wood Sciences Czech University of Life Sciences Prague Prague‐Suchdol Czech Republic; ^2^ Norwegian Institute of Bioeconomy Research (NIBIO) Ås Norway; ^3^ Norwegian Institute for Nature Research (NINA) Oslo Norway; ^4^ TUM School of Life Sciences Technical University of Munich Freising Germany; ^5^ Berchtesgaden National Park Berchtesgaden Germany; ^6^ Regional Plant Health Service – ERSA Friuli Venezia Giulia Italy; ^7^ Swiss Federal Institute for Forest, Snow and Landscape Research WSL Birmensdorf Switzerland; ^8^ Vytautas Magnus University Agricultural Academy Kaunas County Lithuania; ^9^ Forest Research Institute University of Sopron Mátrafüred Hungary; ^10^ Slovenian Forestry Institute Ljubljana Slovenia; ^11^ Forestry Faculty Stefan cel Mare University of Suceava Suceava Romania; ^12^ Department of Agronomy, Food, Natural Resources, Animals and the Environment University of Padua Legnaro PD Italy; ^13^ Forest Research Institute, Bulgarian Academy of Sciences Sofia Bulgaria; ^14^ Forest Research Institute, Sękocin Stary Raszyn Poland; ^15^ Institute for Forest Protection Julius Kühn Institute (JKI) ‐ Federal Research Centre for Cultivated Plants Quedlinburg Germany; ^16^ Faculty of Forest Sciences and Forest Ecology Georg‐August‐University Göttingen Germany; ^17^ Department of Biogeochemical Processes Max Planck Institute for Biogeochemistry Jena Germany; ^18^ Austrian Research Centre for Forests ‐ BFW Vienna Austria; ^19^ Biogeco INRAE, University of Bordeaux Cestas France; ^20^ Department of Ecology Swedish University of Agricultural Sciences (SLU) Uppsala Sweden; ^21^ Slovenia Forest Service Ljubljana Slovenia; ^22^ Natural Resources Institute Finland Joensuu Finland; ^23^ Faculty of Forestry University of Belgrade Belgrade Serbia; ^24^ Faculty of Forestry and Wood Technology Mendel University in Brno Brno Czech Republic; ^25^ National Institute for Research and Development in Forestry Marin “Drăcea” Voluntari Romania; ^26^ Potsdam Institute for Climate Impact Research (PIK) Potsdam Germany; ^27^ European Forest Institute Bonn Germany; ^28^ Croatian Forest Research Institute Jastrebarsko Croatia; ^29^ Nature Research Center Vilnius Lithuania; ^30^ Department of Forest Protection and Forest Protection Service National Forest Centre Banská Štiavnica Slovakia; ^31^ Natural Resources Institute Finland Helsinki Finland

**Keywords:** climate change, ecosystem adaptation, forest disturbance, forest insect herbivores, host tree types, insect feeding guilds

## Abstract

Ongoing shifts in climate and land use have altered interactions between trees and insect herbivores, changing biotic disturbance regimes. However, as these changes are complex and vary across host species, insect taxa, and feeding guilds, they remain poorly understood. We compiled annual records of forest insect disturbance from 15 countries in temperate and boreal Europe, spanning the period from 2000 to 2022. The dataset comprises 1361 time series characterizing the dynamics of 50 herbivorous insects. We used this dataset to test whether insect disturbance has systematically changed during the 23‐year period across host trees and feeding guilds, whether it varies along latitudinal and climatic gradients, and whether synchrony exists among species in the same guild or among species sharing the same host. Since 2000, borer disturbance was predominantly concentrated on gymnosperms, while defoliators impacted gymnosperms and angiosperms more evenly. While 85.8% of gymnosperm disturbance was inflicted by a single species, *Ips typographus*, the majority of disturbances to angiosperms were caused by six different species. Borer impact on gymnosperms has increased in the 21st century, while defoliator impact has decreased across both clades. In contrast to diverging temporal trends, disturbance was consistently greater in warmer and drier conditions across feeding guilds and host types. We identified significant synchrony in insect disturbance within host types and feeding guilds but not between these groups, suggesting shared drivers within guilds and host types. Increasing insect disturbance to gymnosperms may catalyze adaptive transformations in Europe's forests, promoting a shift from historical conifer‐dominated management to broadleaved trees, which are less affected by insect herbivores. Our findings reveal a diversity of trends in insect herbivory, underscoring the need to strengthen monitoring and research in order to better understand underlying mechanisms and identify emerging threats that may not be apparent in currently available data.

## Graphical abstract

Across temperate and boreal Europe, tree mortality caused by insects has risen during the 21st century, yet interactions among insect species and their host trees are complex. While mortality from wood‐boring insects in conifers has increased, defoliation caused by multiple moth species has declined. Regardless of insect or host type, mortality was higher in warmer, drier regions, signaling elevated future risks. Divergent disturbance trends and unequal exposure among tree species remain insufficiently addressed in European adaptation strategies and call for improved monitoring and data sharing to detect emerging risks and guide ecosystem management.

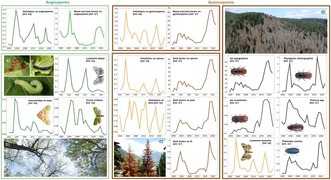

## 1 Introduction

Disturbances play an integral role in natural forest dynamics and the maintenance of ecosystem functioning and biodiversity (Seidl and Turner 2022; Viljur et al. 2022). However, disturbances also can severely impact valued ecosystem services, such as timber production, carbon sequestration, protection against gravitational hazards, regulation of water cycles, and soil protection (Caduff et al. 2022; Lecina‐Diaz et al. 2024; Thom et al. 2017). For example, natural disturbances have significantly contributed to the reduction of carbon sinks in forests globally, potentially turning them into a temporary net source of carbon to the atmosphere (Kurz et al. 2008; Nabuurs et al. 2013; Pugh et al. 2019).

Biotic disturbances, that is, pulses of tree mortality caused by herbivorous insects, pathogens, and vertebrates (Kautz et al. 2017), are among the most impactful disturbances in forests, affecting tens of millions of hectares globally (FAO 2022; van Lierop et al. 2015). In Europe, biotic agents were the cause of 25% of the total growing stock disturbed between 1950 and 2019, and this proportion has increased distinctly in recent years (Forzieri et al. 2023; Patacca et al. 2023). The extent of biotic disturbance has recently even exceeded that of abiotic disturbance in Europe, which constitutes a novel forest disturbance regime for the continent (Patacca et al. 2023). The most impactful feeding guilds are cambio‐ and xylophagous insects feeding on woody tissue (e.g., bark beetles) and phyllophagous insects feeding on tree foliage (e.g., lepidopteran defoliators). For example, a series of drought‐induced outbreaks of the European spruce bark beetle (*Ips typographus*) has affected tens of millions of cubic meters of growing stock in Europe during the recent decade (Hlásny, König, et al. 2021; Hlásny, Zimová, et al. 2021), resulting in the largest wave of tree mortality in Europe in at least 170 years (Senf and Seidl 2021).

Climate change can strongly amplify disturbance dynamics, with warmer and drier conditions increasing the activity of many forest insects and pathogens (Jactel et al. 2019; Seidl et al. 2017). Climate change can also have indirect effects, such as reducing tree resistance due to drought (Hicke et al. 2016; Stephenson et al. 2019). Populations of insects and pathogens often respond rapidly to climatic change due to their physiological sensitivity to temperature, high mobility, short generation times, and high reproductive potential (Weed et al. 2013). Direct climatic effects on bark beetles, wood‐boring insects, and sap‐feeders are often amplified by indirect effects, mediated through climatic effects on host‐tree susceptibility, while effects on defoliators tend to be predominantly direct (Johnson and Haynes 2023). Phloem‐feeding insects of the genera *Ips* and *Dendroctonus* have responded to climate change with remarkable intensity, causing devastating outbreaks across the northern hemisphere in recent decades (Bentz et al. 2010; Hlásny, Zimová, et al. 2021; Kärvemo et al. 2023; Kurz et al. 2008). In contrast, evidence suggests that the impact from defoliators may decline and population cycles may collapse for some species (Allstadt et al. 2013; Ims et al. 2008), possibly due to increasing phenological asynchrony between insect and host as well as altered pest–pathogen interactions. Examples are the possible collapse of long‐term population cycles of larch budmoth (*Zeiraphera griseana*) across the European Alps caused by climate warming (Johnson et al. 2010), and the expansion of the invasive entomopathogen *Entomophaga maimaiga* to Europe, affecting spongy moth ( *Lymantria dispar* ) populations (Holuša et al. 2021; Zúbrik et al. 2018). At the same time, some defoliators, such as the pine processionary moth (*Thaumetopoea pityocampa*) and the oak processionary moth (*Thaumetopoea processionea*), are expanding their range in response to climate warming (Godefroid et al. 2020; Netherer and Schopf 2010; Roques et al. 2015).

Changing herbivory patterns from forest insects interact with the ongoing transformation of European forests, aimed at sustaining ecosystems while addressing society's evolving demands for ecosystem services and products (Hetemäki et al. 2022; Sousa‐Silva et al. 2018). These interactions can align with or counteract European climate, biodiversity, and bioeconomy policies. For example, the ongoing reduction in coniferous forests and promotion of climate‐adapted broadleaved species (Hlásny et al. 2014; Lindner et al. 2010) is accelerated by the increase in disturbances caused by bark beetles colonizing *Picea* and *Pinus* species (George et al. 2022; Hlásny, Zimová, et al. 2021; Jaime et al. 2022); disturbances may thus catalyze adaptation (Seidl et al. 2024; Thom et al. 2017). At the same time, decreasing insect herbivory in angiosperms (Haynes et al. 2014) may facilitate the spread of drought‐tolerant broadleaved species. Nevertheless, the consideration of biotic disturbance trends remains a missing element in many climate change adaptation strategies, which to date largely focus on abiotic factors such as shifting climatic niches of tree species (Hanewinkel et al. 2012; Wessely et al. 2024) and changing productivity patterns (del Castillo et al. 2022).

Although biotic disturbances can severely affect our ability to implement forest‐related policies, our capacity to detect ongoing changes at broad spatial scales remains limited. Among other factors, this is due to the focus of monitoring systems on a few prominent species, the lack of international coordination in survey activities and data sharing, and challenges in disturbance attribution in remote sensing‐based methods to particular agents (Hlásny, Perunová, et al. 2025; Kautz et al. 2017; Senf et al. 2017; Stahl et al. 2023). To address these knowledge gaps, we compiled a novel database of forest disturbance surveys conducted by national forestry agencies across large parts of temperate and boreal Europe, covering the years 2000–2022 (Hlásny, Modlinger, et al. 2025). Although the spatial resolution of these data is coarse, ranging from county‐level administrative regions to entire countries, the temporal resolution is consistent (annual) and the biological level of detail is unique, with disturbance attributed to individual insect species. The database contains only information on native insect species feeding on native tree species.

Due to the lack of coordination and the use of diverse practices among national forest disturbance survey programs in Europe, our first objective was to apply data preprocessing and harmonization to facilitate a consistent Europe‐wide assessment. Based on this harmonized dataset, we aimed to quantify disturbance levels across major feeding guilds (i.e., bark and wood borers, defoliators), host trees, and insect species. We note that the term ‘disturbance’ is used here to broadly refer to both tree mortality caused by bark borers, expressed in cubic meters of affected timber in monitoring, and defoliation caused by defoliating insects, which results in reduced tree vitality but not necessarily tree death and is expressed as area (hectares) affected by defoliation. A subsequent objective was to determine temporal trends, assess the degree of spatial synchrony in disturbances, and quantify the response of disturbance levels to prevalent climatic conditions across main insect species, host trees, and feeding guilds.

We addressed these objectives by testing the following hypotheses: (1) Insect herbivory on gymnosperms has been increasing since 2000 due to the effects of climate change that weaken tree defense and change insect voltinism and overwintering success (Pureswaran et al. 2018; Huang et al. 2020; Singh et al. 2024). By contrast, herbivory trends on angiosperms are more variable owing to their more diverse and complex defense mechanisms. (2) Levels of forest insect disturbance exhibit a consistent decreasing trend with latitude due to increasing thermal constraints on insect development (Liu et al. 2024). (3) Insect disturbance is higher under warmer and drier conditions compared to cooler and wetter climates across all insect species, insect feeding guilds, and host trees (Potterf et al. 2025; Pureswaran et al. 2018), with this pattern being more pronounced in borers than in defoliators. (4) Disturbances caused by insects of the same feeding guild or feeding on the same host tree are more temporally synchronized than disturbances across guilds and hosts because they respond similarly to regional climate anomalies or share natural enemies (Peltonen et al. 2002; Senf and Seidl 2017). Finally, building on our findings, we discuss how trends in insect disturbance align with forest transformation efforts and adaptation targets in the European Union.

## 2 Materials and Methods

**FIGURE 1:**
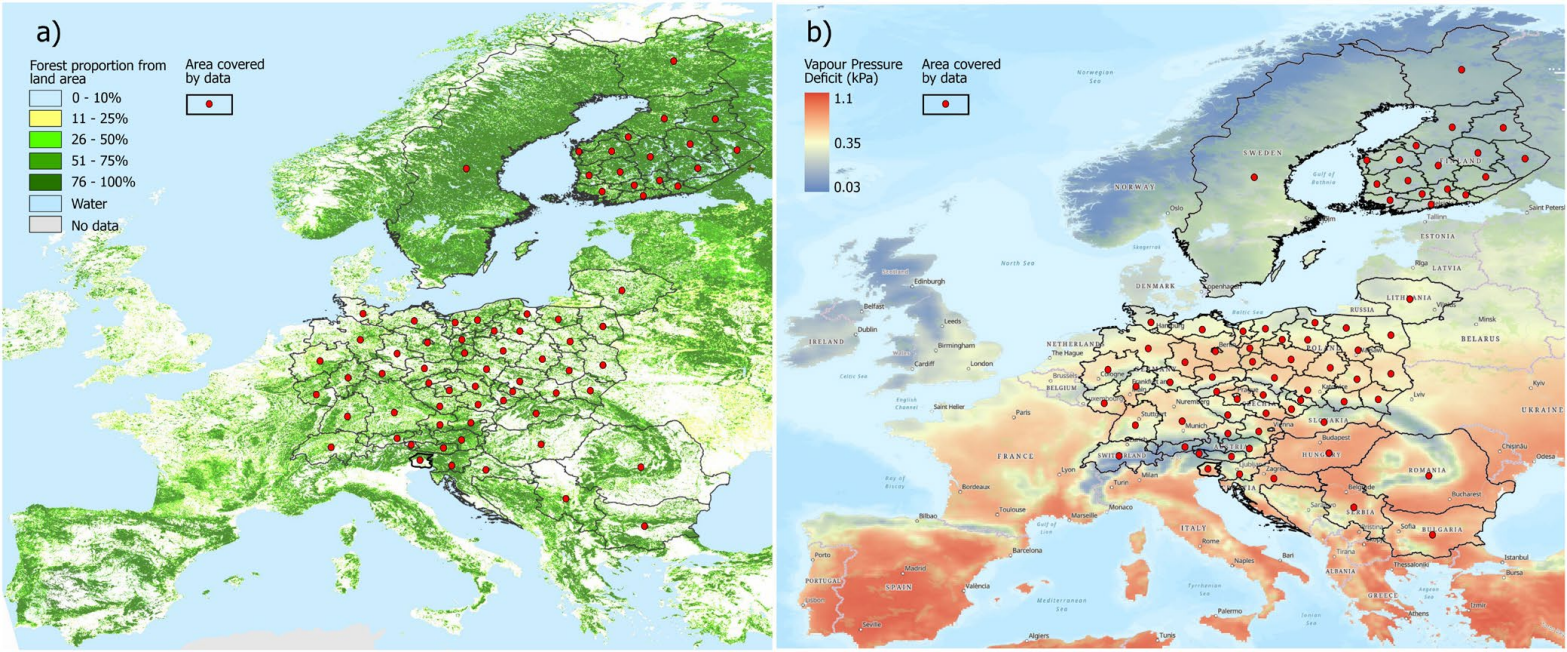
Europe‐wide coverage of insect disturbance data. Each dot represents the centroid of a country or administrative region (polygons) for which species‐specific time series were compiled (a). Source of the forest map: Schuck et al. (2002). Distribution of mean annual Vapor Pressure Deficit values (average for 2000–2022) across Europe used as a predictor of insect disturbance levels (b). Source of climate data: ISIMIP Data Team (2025). Map lines delineate study areas and do not necessarily depict accepted national boundaries.

To identify temporal and geographical trends in forest disturbance caused by different insect species and species groups (Table 1), we collected annual time series covering multiple countries and sub‐national administrative units of the level NUTS2 (Nomenclature of Territorial Units for Statistics, EC 2024) across Europe, with the prevalent length of 23 years (Figure 1). To account for differences in forest conditions among spatial units and to explain observed patterns in insect disturbance, we compiled data on tree species abundance and climatic characteristics for each spatial unit. To address large‐scale geographical trends in the data and account for spatial autocorrelation, we included geographic coordinates (longitude and latitude) of the centroid of each country or NUTS2 region as additional predictor variables (Figure 1).

**TABLE 1:** The species groups of insect herbivores analyzed.

Hierarchical level	Feeding guilds/host species groups	Insect species or aggregated categories	Countries	Number of time series
1	Borers on gymnosperms	Borers on pine, Borers on spruce, Borers on larch, *Dendroctonus micans* , *Ips acuminatus*, *Ips amitinus*, *Ips cembrae*, *Ips duplicatus*, *Ips sexdentatus* , *Ips typographus*, *Phaenops cyanea*, *Pissodes pini*, *Pityogenes bidentatus* , *Pityogenes chalcographus*, *Pityokteines* spp., *Polygraphus poligraphus*, *Tetropium gabrieli*, *Tetropium* spp., *Tomicus* spp., Siricinae	AT, BG, CZ, DE, FI, HR, HU, CH, IT, LT, PL, RO, RS, SE, SI, SK	616/328
1	Borers on angiosperms	*Agrilus biguttatus*, *Agrilus viridis*, *Scolytus scolytus* , *Scolytus intricatus*, *Scolytus ratzeburgii*	CZ, DE, LT, PL	167/73
1	Defoliators on gymnosperms	*Bupalus piniarius*, *Cephalcia* sp., *Dendrolimus pini*, *Diprion* sp., *Lymantria monacha*, *Neodiprion sertifer* , *Panolis flammea*, *Pristiphora abietina*, *Thaumetopoea pityocampa*, *Zeiraphera griseana*	AT, BG, CZ, DE, HR, LT, PL	320/125
1	Defoliators on angiosperms	Defoliators on angiosperms, Defoliators on oaks, Geometridae on oaks, *Clostera anastomosis*, *Erannis defoliaria*, *Lymantria dispar* , *Melolontha* spp., *Operophtera* sp., *Thaumetopoea processionea*, *Tortrix viridana*	AT, BG, CZ, DE, HR, HU, LT, PL, RO, RS, SK	247/118
2	Borers on *A. alba*	*Pityokteines* spp., *Cryphalus* sp., *Pissodes* sp.	AT, CZ, DE, HR, SL	40/21
2	Borers on *P. abies*	Borers on spruce, *Dendroctonus micans* , *Ips amitinus*, *Ips duplicatus*, *Ips typographus*, *Pityogenes chalcographus*, *Polygraphus poligraphus*, *Tetropium* spp., Siricinae	AT, BG, CZ, DE, FI, HR, HU, CH, IT, LT, PL, RO, RS, SE, SI, SK	232/130
2	Defoliators on *P. abies*	*Cephalcia* sp., *Lymantria monacha*, *Pristiphora abietina*, *Zeiraphera griseana*	CZ, DE, LT, PL	119/53
2	Borers on *P. sylvestris*	Borers on pine, *Ips acuminatus*, *Ips sexdentatus* , *Phaenops cyanea*, *Pissodes pini*, *Pityogenes bidentatus* , *Pityogenes chalcographus*, *Tomicus* spp.	AT, BG, CZ, DE, HR, LT, PL, SI, SK	263/144
2	Defoliators on *P. sylvestris*	*Bupalus piniarius*, *Dendrolimus pini*, *Diprion* sp., *Neodiprion sertifer* , *Panolis flammea*, *Thaumetopoea pityocampa*	AT, BG, CZ, DE, LT, PL	201/72
3	*Ips typographus* (borer on *P. abies* )	*Ips typographus*	AT, BG, CZ, DE, FI, HR, HU, CH, IT, LT, PL, RO, RS, SE, SI, SK	98/74
3	*Phaenops cyanea* (borer on *P. sylvestris* )	*Phaenops cyanea*	AT, CZ, DE, LT, PL	56/42
3	*Tomicus* spp. (borers on *P. sylvestris* )	*Tomicus minor*, *Tomicus piniperda*	AT, CZ, DE, LT, PL	56/42
3	*Ips acuminatus* (borer on *P. sylvestris* )	*Ips acuminatus*	BG, CZ, DE, LT, PL, SK	49/26
3	*Pityogenes chalcographus* (borer on *P. abies* )	*Pityogenes chalcographus*	AT, DE, IT, LT, SI, SK	28/23
3	*Tortrix viridana* (defoliator on *Quercus* spp.)	*Tortrix viridana*	BG, DE, LT, PL, RO	35/25
3	Geometridae on oaks (defoliator on *Quercus* spp.)	Geometridae on oaks	BG, DE, LT, PL, RO, HR	36/22
3	*Lymantria dispar* (defoliator on angiosperms)	*Lymantria dispar*	AT, BG, CZ, DE, HR, HU, LT, RO, RS, SK	45/17
3	*Lymantria monacha* (defoliator on gymnosperms)	*Lymantria monacha*	CZ, DE, LT, PL	32/26

*Note:* Species or species groups, countries, and the number of time series included in analyses are indicated. In the “number of series” column, the first number indicates the total number of time series available. The second number indicates the number of time series with more than six non‐zero values, which were used for trend analysis. The combination of single species and higher‐level categories (e.g., borers on *Pinus sylvestris* ) within the same insect category stems from the heterogeneous structure of data reported by the participating countries. Country codes: AT: Austria, BG: Bulgaria, CH: Switzerland, CZ: Czechia, DE: Germany, FI: Finland, HR: Croatia, HU: Hungary, IT: Italy, LT: Lithuania, PL: Poland, RO: Romania, RS: Serbia, SE: Sweden, SI: Slovenia, SK: Slovakia.

Given the high risk of spatial, temporal, and spatiotemporal autocorrelation, which can inflate degrees of freedom, we used mean host tree abundance and mean climatic conditions over the study period rather than annual time series of these predictors. Hence, our analysis focuses on detecting temporal trends within disturbance series and is designed to reveal spatial responses to climatic and geographical gradients, rather than to explain year‐to‐year variation in forest insect disturbance.

### 2.1 Forest Disturbance Data

Data used in this study were compiled from national forest disturbance survey programs operated by various governmental agencies across Europe (Hlásny, Modlinger, et al. 2025). These data are typically collected as part of forest disturbance management activities, such as salvage and sanitation harvesting of dead or infested trees, or aerial treatment of infested stands (e.g., EC and JRC 2021; Hlásny, Perunová, et al. 2025; Kunca et al. 2019). Although country‐specific approaches differ, a general data acquisition process is as follows: Disturbance is typically recorded directly in the field, during harvesting operations in the case of agents causing tree mortality (e.g., bark beetles), or through visual inspection, sometimes supported by aerial surveys, in the case of defoliators. Surveys are typically conducted by local forestry personnel. In most countries, these personnel are trained to estimate the affected volume or area and to attribute disturbance to major agents, including insects. When unusual or unclear disturbance occurs, experts from local forestry agencies assist in agent identification, including sample collection for laboratory analysis when needed. After submitting field records to national data centers, completeness and quality control procedures are applied by experts, evaluating logical consistency, plausibility of interannual and regional variation, and other relevant aspects of the dataset. Such procedures apply to all countries except Finland and Sweden, where data consistency and completeness are lower (Appendix B).

The compiled forest disturbance data were collected from 15 countries within Europe's temperate and boreal zones, and from a single administrative district in Italy (Figure 1, Table 1). Collectively, the dataset represents 61.5% of the forest area of the European Union plus Switzerland and Serbia, covering 102.75 million hectares of forest. Data primarily cover the period 2000 to 2022, with a time series length of up to 23 sequential years of annual records; however, time series are shorter for some countries (Appendix A). Some countries record time series of annual disturbance levels across the entire country for each insect species, while other countries (Poland, Czechia, Austria, Germany, and Finland) report multiple time series for the countries' administrative regions at level NUTS2 (Figure 1). Each time series reports the annual level of disturbance caused by an individual insect species on a specific host tree species (or, in a few cases, a broader species group, such as *Geometridae on oaks*) in a particular country or NUTS2 region. The final dataset contained 30,327 individual records organized into 1361 time series for 82 spatial units, characterizing insect herbivory for 50 different insect herbivore species (Table 1, Appendix A).

### 2.2 Data Preprocessing

Data collection procedures, disturbance attribution to insect species, and the details of reporting differed somewhat between countries, requiring several preprocessing steps to harmonize the data prior to analysis:

- In most national databases, disturbance caused by defoliators was recorded as the area with noticeable defoliation, while disturbance caused by bark and wood borers was recorded as the volume (m^3^) of dead and/or infested trees. However, some countries reported units of area for all agents, necessitating a conversion from area affected to volume in the case of borers. We used country‐specific conversion factors based on consultations with national experts to homogenize the units of reporting (Appendix B). In Germany, Bulgaria, Finland, Romania and Lithuania, some disturbance was reported in both aerial and volume units, which facilitated conversion between units.
- In some countries, disturbance was recorded across all forest land while in other countries disturbance was only recorded on public forest land. In the latter case, reported disturbance levels were upscaled to the entire country based on the proportion of public forest land relative to total forest land. The situation was opposite in Finland, where forest disturbance data were reported for private forests only (Appendix B).
- In the case of Poland, data were reported at the level of national forest districts which only partly match NUTS2 regions. For consistency, we identified which districts fell within each NUTS region and then aggregated the disturbance values accordingly.
- Although we aimed to acquire data at the level of individual insect species, some countries reported broader categories such as *bark beetles on gymnosperms* or *defoliators on oaks*. For Poland, we used time‐invariant expert‐derived proportions to split these categories and attribute the disturbance to individual species (Appendix B). If the national data providers indicated that a broader group was dominated by a single species (e.g., the category *borers on gymnosperms* is dominated by *I. typographus*), we considered this group in species‐specific analyses. If such an attribution was not possible, these time series were only considered in analyses summarizing disturbance at higher hierarchical levels (Section 2.3, Table 1), but were excluded from species‐level analyses.

### 2.3 Data Aggregation

We structured the identification of Europe‐wide insect disturbance trends into several hierarchical levels, defined by the combination of host trees (species, genus, or higher taxonomic order) and insect feeding guilds (bark and wood borers and defoliators). To facilitate this analysis, each insect species was categorized by its main host tree genus: *Quercus*, *Fagus*, *Larix*, *Picea*, *Pinus*, and *Abies*. For polyphagous species, the most commonly affected host species was used for this classification, based on reports from the literature and consultations with national data providers. For example, all defoliation events attributed to *L. monacha* were assigned to *P. abies* even though the species also feeds on *P. sylvestris* (Bejer 1988; Nakládal and Brinkeová 2015). At a higher taxonomic level of host tree categorization, the data was grouped separately for angiosperms and gymnosperms. Next, each insect was classified as either borer or defoliator. Since disturbance caused by borers was reported in cubic meters and defoliations in hectares, an analysis across these groups (such as summarizing total disturbance to gymnosperms) was not possible. Hence, our analyses used various combinations of host types and feeding guilds, without combining guilds in a single analysis (Table 1). Species‐level analyses were limited to a few species that ranked highly in terms of total impact (Table 3), were represented by at least 15 time series, and were reported from a minimum of four countries. Based on these criteria, we selected nine host tree‐feeding guild combinations and nine single species for detailed analyses (hierarchical level 1 + 2 and 3, respectively, Table 1).

### 2.4 Climatic Data and Data on Host Tree Abundance

To avoid multicollinearity associated with using a large number of climatic variables and indices, we selected mean annual Vapor Pressure Deficit (VPD) as an integrative climatic indicator. VPD effectively captures the dominant continental gradient from warm‐dry to cool‐wet conditions (Nagavciuc et al. 2024) and has been consistently demonstrated as a predictor of vegetation status, including plant defense against biotic stressors (Das et al. 2025; Hartmann et al. 2018). The strong, slightly nonlinear correlation between VPD and temperature (*R* ^2^ 0.92; Appendix E) highlights its utility as a proxy not only for water stress but also for thermal constraints on insect life cycles and distribution. Data used to produce a mean VPD map for the 2000–2022 period across Europe (Figure 1b) were derived from a daily climatological dataset provided by the Inter‐Sectoral Impact Model Intercomparison Project (ISIMIP) Data Team. The dataset is based on the ERA5‐Land reanalysis (Muñoz‐Sabater et al. 2021), which offers hourly global data at a 0.1° spatial resolution from 1950 onwards. For this study, we used daily mean temperature and relative humidity values and calculated daily VPD using the “RHtoVPD” function from the “plantecophys” R package (Duursma 2015). The resulting daily VPD values were then aggregated to compute average annual VPD values for the entire 2000–2022 period (Figure 1b). The VPD values used as a predictor of disturbance levels were calculated as the mean VPD across forested areas within each spatial unit (country or NUTS2 region; Figure 1).

The abundance of host tree species and host tree groups, corresponding to the insect taxa specified in Table 1, was compiled for each spatial unit (Figure 1) using different data sources, primarily National Forest Inventories from the participating countries (see Appendix D and Supporting Information).

### 2.5 Statistical Analyses

Prior to analyses, individual time series on forest disturbance that contained less than six non‐zero values were removed. Next, data were split into 18 nested subsets characterizing selected insect species, host tree groups, and feeding guilds (Table 1). All analyses were conducted using log transformed values (log(*x* + 1)). Even though different non‐linear patterns may be present in the data, the limited length of the available time series and the small number of observed outbreak cycles constrain our options to model such non‐linearities reliably. Therefore, we chose to focus on linear trends, addressing the most parsimonious question: Has tree mortality or defoliation caused by different insect herbivores and feeding guilds on various host trees increased or decreased since 2000?

To identify temporal and geographical disturbance trends for the 18 groups listed in Table 1, we used linear mixed models (glmmTMB; Brooks et al. 2017) in R (R Core Team 2024) to regress variables “Year,” “VPD,” “Longitude,” and “Latitude” on insect disturbance values within species groups. To account for differences in host amount within countries and regions, we included the variable “Host” as an additional predictor (Appendix D). All variables except for “Year” were considered temporally invariant.

The administrative regions for which the data were available (i.e., country or NUTS2 regions, Figure 1) were specified as random effects, thereby accounting for differences in natural conditions and data quality across spatial units. Since higher‐level categories (hierarchical level 1 and 2, Table 1), such as *defoliators on angiosperms* or *borers on spruce* included more than one host or insect species, “Insect species” and “Host species” were also included as random effects. Temporal autocorrelation was accounted for by implementing the AR(1) covariance structure in glmmTMB. Where applicable, and when model performance allowed, AR(1) functions were specified separately within administrative regions and insect species. Zero inflation in the data was accounted for using the formula “ziformula = ~(1|species).” Since some categories required a simpler parametrization of the AR(1) covariance structure, the final model specifications were determined using the “diagnose” function in glmmTMB.

Finally, we aimed to identify disturbance change rates across “Year,” “VPD,” “Longitude,” and “Latitude.” To accomplish this, effect sizes from the linear mixed effects models on the 18 groups were back‐transformed. Since back‐transformation introduces non‐linear patterns to linear model expressions, beta (or slope) values could not be back‐transformed directly. Instead, to obtain the rate of change for untransformed values, disturbance values were predicted for the start and endpoint of the predictor variable ranges using the following formula:

(1) $$R={\left(\frac{\mathrm{end}\;\text{value}}{\text{start value}}\right)}^{1/n}-1$$

For the predictor “Year,” the start and end values correspond to 2000 and 2022. “Latitude” and “Longitude” represent the southernmost to northernmost and westernmost to easternmost locations in the dataset (represented as centroids in Figure 1), respectively. For “VPD,” they refer to the driest to wettest spatial unit (Figure 1).

We did not control for experiment‐wide error levels (e.g., by applying a Bonferroni correction) as each test addressed a distinct hypothesis, and we chose to maintain statistical power in evaluating each analysis independently.

## 3 Results

### 3.1 Disturbance Levels and Patterns

**FIGURE 2:**
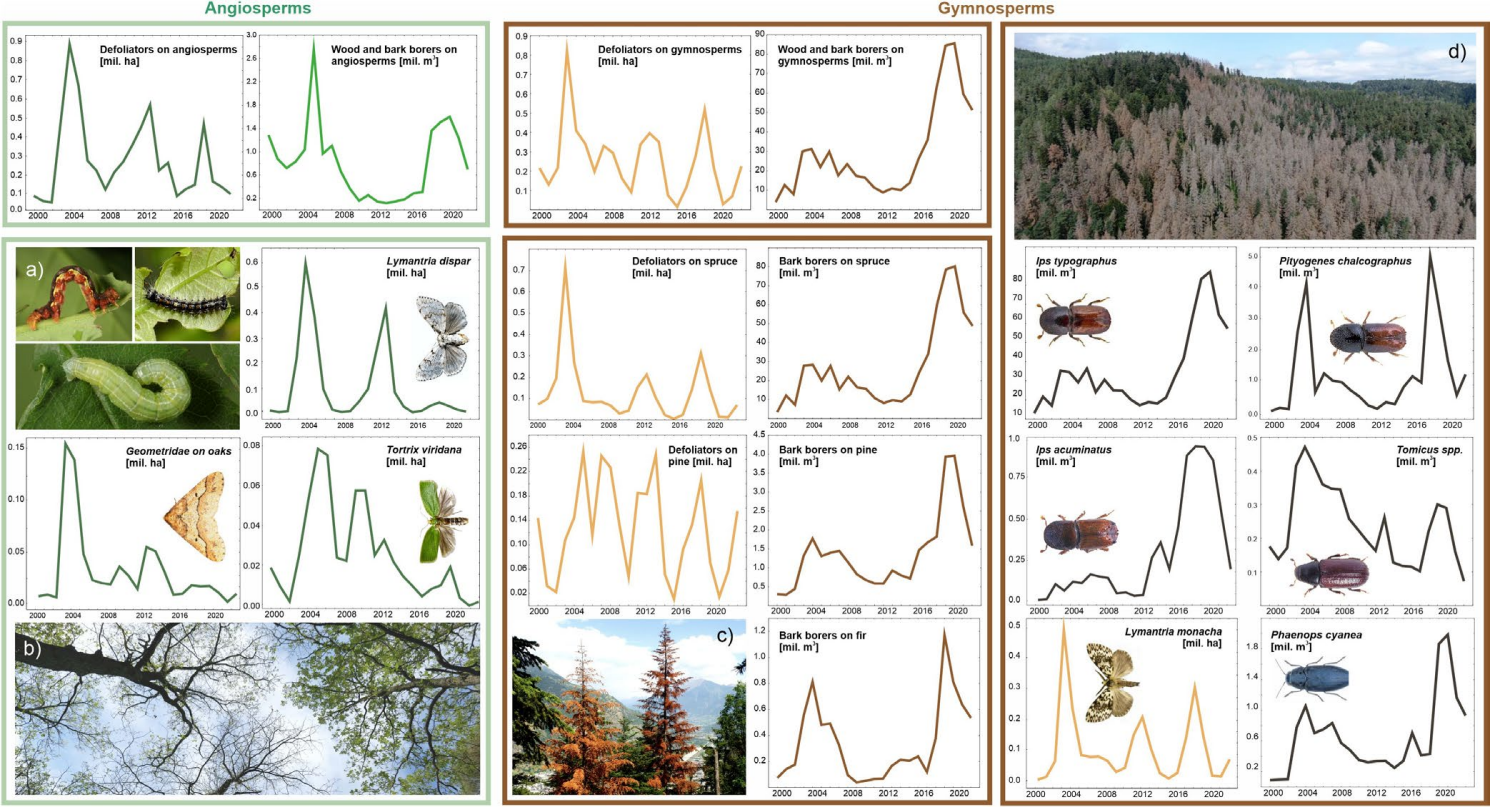
Temporal variation in forest disturbance caused by selected insect species and species groups from 2000 to 2022. The data represents 15 European countries. Note that the y‐axis ranges differ between the plots. Inset images: (a) caterpillars of defoliators *Erannis defoliaria*, *Lymantria dispar* and *Operophtera brumata* (clockwise) (Photo: G. Csóka); (b) Defoliation of *Quercus petraea* trees by *Lymantria dispar* , Central Czechia (Photo: R. Modlinger); (c) Mortality of *Abies alba* in Switzerland following infestation by *Pityokteines* spp. (Photo: S. Blaser); (d) Large‐scale mortality of *Picea abies* following infestation by *Ips typographus*, Bohemian Forest National Park, Czechia (Photo: R. Modlinger). Insect images in the right panel: Forest Protection Service, Czech Republic.

During the period 2000–2022, the most prominent biotic disturbance was caused by borers, which impacted a total of 684 million m^3^ of wood (Table 2). Of this value, 98.4% was disturbance to gymnosperms, and 92.3% was disturbance to *Picea abies* . Temporally, disturbance caused by borers exhibited two distinct peaks, a first peak between 2002 and 2008, and a second, much larger peak after 2015 (Figure 2). In terms of the proportion of annually affected growing stock for each host group (Table 2, Appendix D), borer disturbance was more pronounced in gymnosperms (0.27% year^−1^) than in angiosperms (0.01% year^−1^). Within gymnosperms, *P. abies* experienced the highest relative disturbance levels of 0.53% of growing stock affected per year, whereas disturbance to *Abies alba* and *Pinus sylvestris* was lower, at 0.07% year^−1^ and 0.03% year^−1^, respectively.

**TABLE 2:** Level of tree mortality (in case of borers, m^3^) and defoliation (in case of defoliators, ha) as well as the proportion of host growing stock or area affected annually during the period 2000–2022.

Hierarchical level	Species/species groups	Cumulative impact 2000–2022	Proportion host of growing stock or area affected annually (%)
1	Borers on gymnosperms	673,169,401 m^3^	0.27
1	Borers on angiosperms	10,869,383 m^3^	0.01
1	Defoliators on gymnosperms	5,566,820 ha	0.46
1	Defoliators on angiosperms	6,393,628 ha	0.95
2	Borers on *Abies alba*	7,845,375 m^3^	0.07
2	Borers on *Picea abies*	631,243,712 m^3^	0.53
2	Borers on *Pinus sylvestris*	30,553,861 m^3^	0.03
2	Defoliators on *Picea abies*	2,916,474 ha	0.52
2	Defoliators on *Pinus sylvestris*	2,650,346 ha	0.46
3	*Ips typographus*	586,715,446 m^3^	0.49
3	*Phaenops cyanea*	14,865,369 m^3^	0.02
3	*Tomicus* spp.	5,432,690 m^3^	0.01
3	*Ips acuminatus*	6,845,704 m^3^	0.01
3	*Pityogenes chalcographus*	28,145,602 m^3^	0.02
3	*Tortrix viridana*	641,455 ha	0.46
3	*Geometridae on oaks*	746,724 ha	0.53
3	*Lymantria dispar*	2,390,823 ha	1.70
3	*Lymantria monacha*	2,402,944 ha	0.43

*Note:* The three hierarchical levels indicate (1) feeding guilds, categorized by host type (gymnosperms vs. angiosperms); (2) gymnosperm host genera; and (3) selected insect species.

Disturbance caused by defoliators totaled 11.9 million ha between 2000 and 2022, of which 53.5% was inflicted on angiosperms. Defoliation of angiosperms was mainly associated with *Quercus* spp. (caused mainly by moth species, such as *Tortrix viridana* and *L. dispar* ), while defoliation of gymnosperms was mainly associated with *L. monacha* . We note that defoliation on *P. abies* is likely overestimated and on *P. sylvestris* underestimated, as defoliations caused *L. monacha* , which feeds on both hosts, were attributed to the dominant host *P. abies* , due to lack of precise attribution in the source data. In terms of relative disturbance, an average of 0.46% of the area occupied by gymnosperms and 0.95% of angiosperms were defoliated annually between 2000 and 2022. The most extensive defoliation was caused by *L. dispar* (1.7% year^−1^), while the remaining defoliators affected 0.46%–0.53% of the host area annually (Table 2).

### 3.2 Species Contribution to Total Disturbance

**FIGURE 3:**
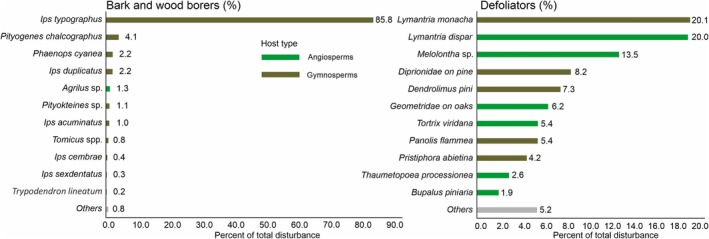
Relative contribution of individual insect herbivores to the total recorded disturbance. The contributions are presented separately for borers and defoliators, differentiated by host type. For polyphagous species, the host type was defined based on the most common host.

Within each feeding guild, the contribution of individual insect species to total disturbance was uneven, with a few species being responsible for most of the disturbance (Figure 3). This pattern was most distinct for borers, with *I. typographus* causing 85.8% of the total disturbance, followed by *Pityogenes chalcographus* (4.1%), *I. duplicatus* (2.2%), and *Phaenops cyanea* (2.2%). Disturbance was distributed more evenly across defoliator species, with *L. monacha* and *L. dispar* being responsible for 20.1% and 20.0% of total disturbance, respectively, followed by *Melolontha* spp. (13.5%) and a broader group of Geometrids on oaks (6.2%), represented by species such as *Erannis defoliaria*, *Operophtera brumata* , and *O. fagata*.

The unevenness in disturbance caused by different species within each feeding guild was even more pronounced when considering host tree types. Among borers, 98.4% of the disturbance occurred on gymnosperms, while the remaining disturbance, caused by borers such as *Agrilus* spp. (1.3%), was on angiosperms. Among defoliators, 53.5% of the disturbance affected angiosperms (notably oaks), while the rest occurred on gymnosperms.

### 3.3 Temporal and Geographical Disturbance Trends

**FIGURE 4:**
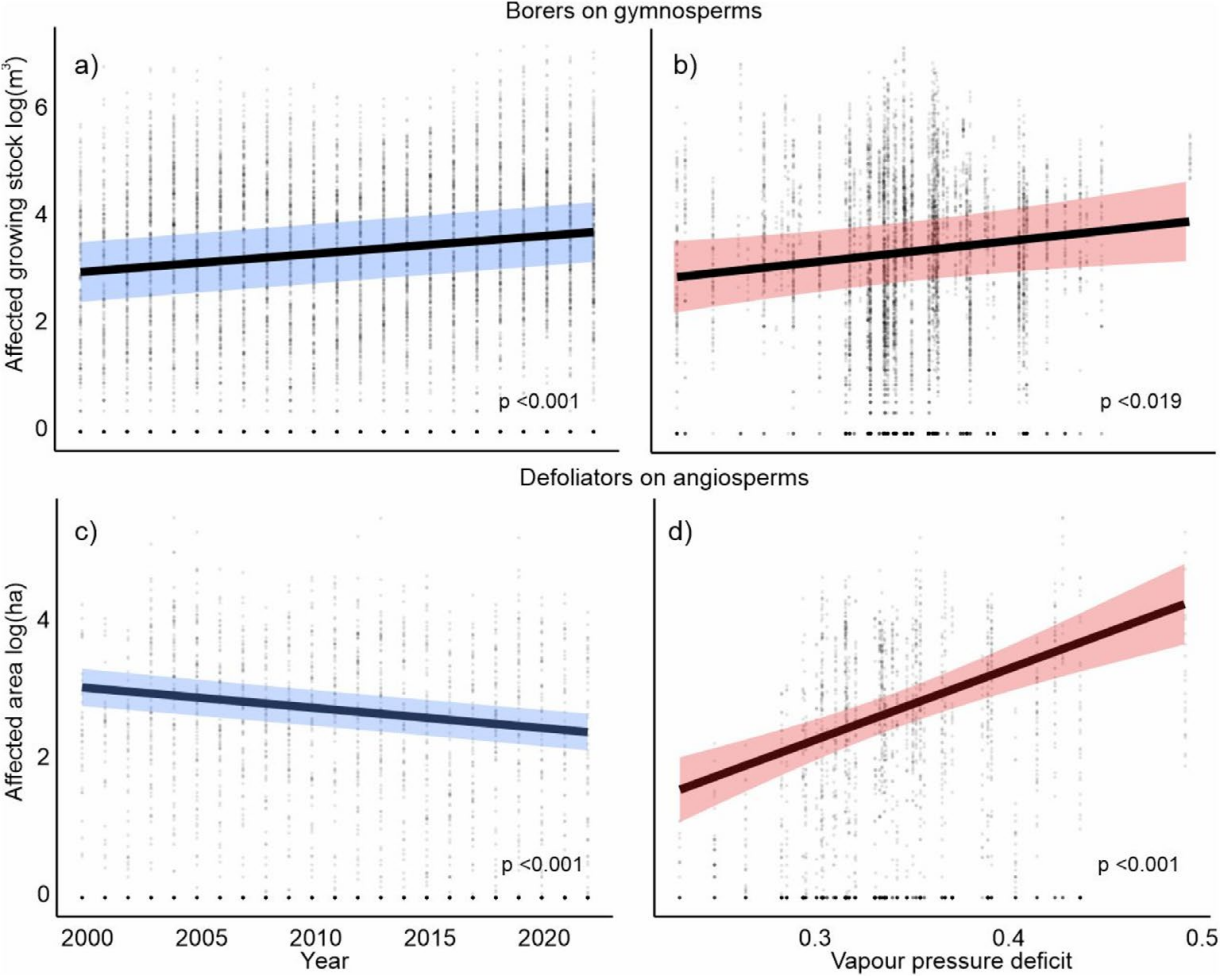
Divergent temporal trends in forest disturbance caused by bark‐ and wood‐boring insects on gymnosperms versus defoliators on angiosperms (a,c), along with a consistent response to vapor pressure deficit (b,d). These patterns are broadly consistent across insect species and their respective host trees within the two major feeding guilds (Figure 5).

**FIGURE 5:**
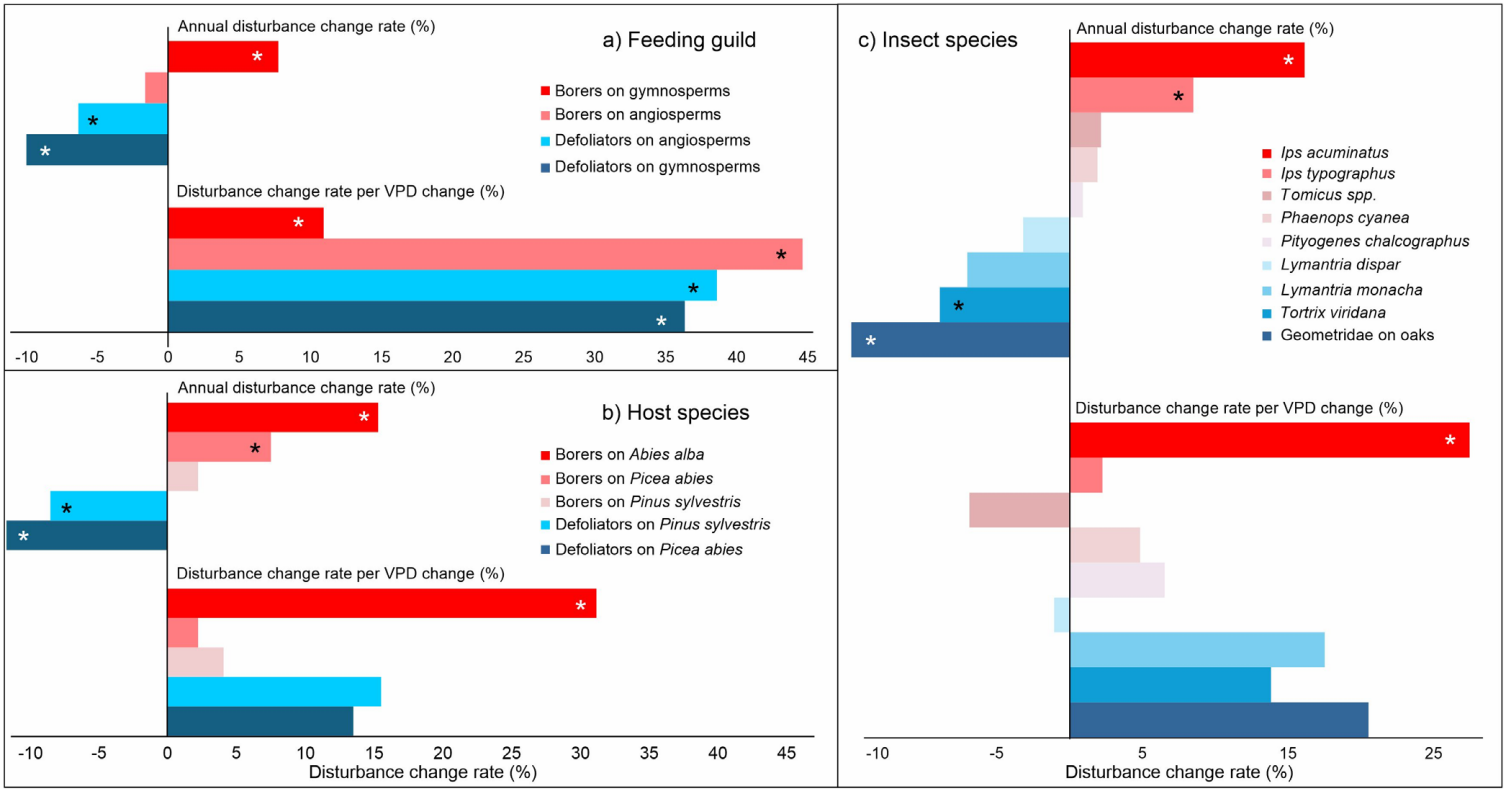
Relative change rates in forest disturbance caused by insect herbivores, shown by: (a) feeding guilds, categorized by host type (gymnosperms vs. angiosperms); (b) gymnosperm host genera; and (c) selected insect species. Each panel presents annual disturbance change rates between 2000 and 2022 (expressed as percent change per year) alongside relative disturbance change rates per unit change in vapor pressure deficit (VPD). In each panel, the species and species groups are organized in descending order based on the annual change rates. An asterisk denotes the statistical significance of the linear trends at *p* < 0.001.

The two feeding guilds, borers and defoliators, exhibited distinctly different temporal trends between 2000 and 2022 (Figures 4 and 5, Table 3, Appendix D). At the same time, we observed a consistently positive relationship between disturbance and the VPD gradient across the guilds.

**TABLE 3:** Temporal and spatial trends in insect disturbance based on linear regressions of log‐transformed data.

Insect group	Annual change rate (%)	*p*	VPD change rate (%)	*p*	Lat. change rate (%)	*p*	Long. change rate (%)	*p*
Borers on gymnosperms	7.77	< 0.001	10.97	0.019	−4.31	0.358	−5.10	0.195
Borers on angiosperms	−1.61	0.303	44.69	< 0.001	26.50	0.093	7.74	0.231
Defoliators on gymnosperms	−9.98	< 0.001	36.39	< 0.001	43.65	0.001	11.45	0.128
Defoliators on angiosperms	−6.31	< 0.001	38.66	< 0.001	36.79	< 0.001	−2.01	0.656
Borers on *A. alba*	15.34	< 0.001	31.22	< 0.001	−12.09	0.650	−33.04	< 0.001
Borers on *P. abies*	7.54	< 0.001	2.24	0.636	−12.09	0.007	−8.21	0.04
Borers on *P. sylvestris*	2.25	0.162	4.10	0.589	3.61	0.767	26.47	0.001
Defoliators on *P. abies*	−11.69	< 0.001	13.55	0.337	203.68	< 0.001	−4.72	0.636
Defoliators on *P. sylvestris*	−8.50	< 0.001	15.56	0.303	3.87	0.826	5.79	0.557
*Ips typographus*	8.49	< 0.001	2.24	0.619	−13.04	0.002	−8.75	0.021
*Phaenops cyanea*	1.90	0.085	4.83	0.436	25.13	0.053	1.84	0.708
*Tomicus* spp.	2.15	0.234	−6.89	0.235	−0.30	0.978	7.28	0.19
*Ips acuminatus*	16.13	< 0.001	27.45	0.011	13.23	0.340	52.02	< 0.001
*Pityogenes chalcographus*	0.90	0.409	6.52	0.181	−9.69	0.193	5.93	0.286
*Tortrix viridana*	−8.92	< 0.001	13.81	0.089	21.01	0.063	−10.01	0.013
Geometridae on oaks	−15.63	< 0.001	20.51	0.115	−6.84	0.550	−9.30	0.165
*Lymantria dispar*	−3.20	0.142	−1.07	0.892	−17.09	0.088	8.80	0.112
*Lymantria monacha*	−7.04	0.339	17.50	0.35	181.49	< 0.001	−14.75	0.112

*Note:* Results are presented for selected insect species and species groups, defined by host trees and feeding guilds. Rates of change along temporal (Year), climatic (Vapor Pressure Deficit, VPD), and geographical (Latitude, Longitude) gradients are presented. Details of the underlying linear regression results are provided in Appendix D.

Disturbance caused by borers on gymnosperms increased at an average annual rate of 7.8% (*p* < 0.001). The increase was most pronounced for disturbance to *A. alba* (15.3% year^−1^, *p* < 0.001), followed by *P. abies* (7.5% year^−1^, *p* < 0.001), and *P. sylvestris* (2.3% year^−1^, *p* = 0.16). At the species level, the most distinct increase was observed for *I. acuminatus* (16.1% year^−1^, *p* < 0.001), though the absolute amount of disturbance caused by this species was low, accounting for only 1% of the total borer disturbance to gymnosperms (Figure 3). For the dominant species, *I. typographus*, the annual increase rate was 8.5% (*p* < 0.001). The remaining borers showed insignificant yet positive trends (Figure 4).

Disturbance caused by defoliators decreased over time at an annual rate of −10.0% (*p* < 0.001) for gymnosperms and −6.3% (*p* < 0.001) for angiosperms. The decrease was most pronounced for *P. abies* (−11.7% year^−1^, *p* < 0.001), followed by *P. sylvestris* (−8.5% year^−1^, *p* < 0.001). At the insect species level, the most distinct decrease was observed for Geometridae on oaks (−15.6% year^−1^, *p* < 0.001), which accounted for 6.2% of all defoliation, and *T. viridana* (−8.9% year^−1^, *p* < 0.001), which accounted for 5.4% of all defoliation (Figure 3). Defoliation by *L. monacha* also exhibited a downward insignificant trend of −7.0% per year (*p* = 0.300).

In contrast to the variable temporal trends, we found a consistently positive association between disturbance levels and VPD across major feeding guilds (Figure 5a, Table 3), indicating higher disturbance in warmer and drier locations. All effects were statistically significant at the level of feeding guilds, with effect sizes ranging from 11.0% to 44.7% per 0.1 kPa change in VPD. At the level of individual host and insect species, the relationships were mostly positive but not statistically significant (Figure 5b,c), except for *A. alba* and *I. acuminatus* , which exhibited a significantly positive response. The magnitude of change at the species and host level was smaller than that observed at the guild level.

Latitudinal and longitudinal trends varied among species and species groups and did not reveal consistent patterns (Table 3). An exception was a negative latitudinal trend in some species and species groups, suggesting the potential for future northward shifts in disturbance. This pattern was particularly evident in borers affecting *A. alba* , where disturbance decreased by 57.5% with each degree of latitude (*p* = 0.01). At the insect species level, a similar pattern was identified for *I. typographus* (−13.0%, *p* = 0.002), *P. chalcographus* (−9.7%, *p* = 0.19), and *L. dispar* (−17.1%, *p* = 0.09). The longitudinal pattern was less clear, with varying responses within species groups.

Finally, disturbance response to the amount of a given host species or host species group was plausible across all insect species, with disturbance increasing with host growing stock or area (Appendix D). Since this variable was mainly included to account for differences in host availability across countries, we do not interpret the coefficients further here.

### 3.4 Synchrony in Disturbance Trends

Correlations between pairs of time series were non‐negative within each of the three hierarchical levels analyzed (insect feeding guild, host tree genus, and insect species) (Tables 4, 5, 6). At the feeding guild level, significant correlations were found between borers on gymnosperms and borers on angiosperms (*r* = 0.21), as well as between defoliators on gymnosperms and defoliators on angiosperms (*r* = 0.20). These results indicate synchrony within feeding guilds, but not between them (Table 4). At the host tree level, we identified high and significant positive correlations among borers on *P. abies* , *P. sylvestris* , and *A. alba* , with correlation coefficients ranging from 0.63 to 0.88 (Table 5). In contrast, all correlations between defoliators on *P. abies* and *P. sylvestris* were insignificant.

**TABLE 4:** Pearson correlations between time series of total disturbance recorded within the investigated geographical domain for the main insect feeding guilds.

Disturbance series	Borers on gymnosperms	Borers on angiosperms	Defoliators on gymnosperms	Defoliators on angiosperms
Borers on gymnosperms	1.00			
Borers on angiosperms	0.21	1.00		
Defoliators on gymnosperms	0.00	0.02	1.00	
Defoliators on angiosperms	0.00	0.04	0.20	1.00

*Note:* Red values indicate statistical significance at *α* < 0.001.

**TABLE 5:** Pearson correlations between time series of total disturbance recorded within the investigated geographical domain for the different insect feeding guilds defined by host tree genus.

Disturbance series	Borers on *Abies alba*	Borers on *Picea abies*	Borers on *Pinus sylvestris*	Defoliators on spruce	Defoliators on *P. sylvestris*
Borers on *A. alba*	1.00				
Borers on *P. abies*	0.63	1.00			
Borers on *P. sylvestris*	0.73	0.88	1.00		
Defoliators on *P. abies*	0.04	0.00	0.00	1.00	
Defoliators on *P. sylvestris*	0.03	0.04	0.03	0.02	1.00

*Note:* Red values indicate statistical significance at *α* < 0.001.

The correlation pattern among individual insect species was more complex, with most of the significant correlations occurring between pairs of borer species such as *I. typographus*, *P. cyanea* , *P. chalcographus*, and *Tomicus* spp., and between pairs of defoliator species such as *T. viridana*, *L. monacha* , *L. dispar* , and Geometrid species (Table 6). However, *Tomicus* spp. (borers on *P. sylvestris* ) were significantly correlated with all defoliators, and *P. chalcographus* (borer on *P. abies* ) was significantly correlated with *L. monacha* (a defoliator affecting mostly gymnosperms).

**TABLE 6:** Pearson correlations between the time series of total forest disturbance recorded within the investigated geographical domain for different insect species.

Disturbance series	*I. typographus*	*P. cyanea*	*Tomicus* spp.	*I. acuminatus*	*P. chalcographus*	*T. viridana*	Geometridae on oaks	*L. dispar*	*L. monacha*
*Ips typographus*	1.00								
*Phaenops cyanea*	0.73	1.00							
*Tomicus* spp.	0.01	0.15	1.00						
*Ips acuminatus*	0.68	0.36	0.01	1.00					
*Pityogenes chalcographus*	0.34	0.23	0.20	0.28	1.00				
*Tortrix viridana*	0.07	0.00	0.44	0.17	0.01	1.00			
Geometridae on oaks	0.02	0.00	0.38	0.07	0.15	0.19	1.00		
*Lymantria dispar*	0.03	0.00	0.34	0.06	0.06	0.31	0.61	1.00	
*Lymantria monacha*	0.01	0.01	0.20	0.00	0.39	0.02	0.56	0.20	1.00

*Note:* Red values indicate statistical significance at *α* < 0.001.

## 4 Discussion

Forest insect disturbances respond strongly to climate and land‐use change and vary across geographical gradients, yet it remains unclear how these trends manifest across different insect species and their host trees. We revealed contrasting temporal trends between major insect feeding guilds and between gymnosperms and angiosperms across Europe, and consistently higher disturbance levels in warmer and drier regions. Apart from novel ecological insights, these findings carry important implications for adaptation and bioeconomy strategies in Europe—for example, they can inform tree species selection not only with respect to current and future site suitability but also in relation to evolving biotic risks. These findings are based on a novel disturbance dataset with several unique features that can effectively complement existing datasets and support future research on forest disturbance dynamics across Europe. Below, we explore the drivers of these trends, their implications for ecosystem management, and the limitations of our newly developed dataset.

### 4.1 Temporal Disturbance Trends

Consistent with our initial hypothesis, we found a clear increasing trend in borer disturbance to gymnosperms across all studied host species in the 21st century. In contrast, both borer and defoliator disturbance to angiosperms declined, with this pattern consistently observed across all examined insect species. Although such patterns have been reported previously for specific regions for both borers (e.g., Hallas et al. 2024; Hlásny, Zimová, et al. 2021; Kärvemo et al. 2023) and defoliators (e.g., Johnson et al. 2010), we provided the first comprehensive assessment confirming these contrasting trends across temperate and boreal biomes of Europe.

The disturbance caused by borers in both gymnosperms and angiosperms occurred in two distinct mortality peaks culminating in 2005 and 2019, closely linked to the extreme drought events of 2003 and 2018 (Schuldt et al. 2020; Peters et al. 2020). A similar mortality pattern was identified by George et al. (2022) using data from the International Cooperative Program on Assessment and Monitoring of Air Pollution Effects on Forests (ICP Forests; Lorenz 1995), although their assessment considered a broader range of mortality causes. While processes behind the 2005 disturbance peak were complex (e.g., Rouault et al. 2006), the peak in 2019 was, to a large extent, attributed to the wave of drought‐induced outbreaks of *I. typographus*, which affected countries throughout Europe, including Germany (Obladen et al. 2021), Czechia (Hlásny, König, et al. 2021), Austria (Hallas et al. 2024), and Sweden (Kärvemo et al. 2023).

Although the increasing trend in insect disturbance to *P. sylvestris* was not statistically significant (annual increase of 2.25%), the trend aligns well with the widely reported mortality of the species over the last two decades, which has gradually extended from the species' arid distributional limits to the center of its climatic niche (Bose et al. 2024). The disturbance on *P. sylvestris* was mainly caused by *P. cyanea* , *I. acuminatus* , and *Tomicus* spp., which all have been found to increase their impacts under increasingly hot and dry conditions (Chinellato et al. 2014; Grégoire and Evans 2007; Hlávková and Doležal 2022; Papek et al. 2024). Consistent with our results, George et al. (2022) found a positive yet insignificant temporal trend in *P. sylvestris* mortality in the ICP Forests monitoring network.

A surprising finding was the sharp increase in disturbance caused by borers affecting *A. alba* (an annual increase of 15.3%), primarily caused by species of the *Pityokteines* family. Although this disturbance accounted for only 1.1% of the total disturbance to gymnosperms (reflecting the modest proportion of *A. alba* in the European forests of around 5% of forest area), this trend deserves attention. Mortality dynamics of *A. alba* are understudied (but see, for example, Knížek et al. 2023; Oliva and Colinas 2007; Podlaski et al. 2020), while the current and future ecological importance of the species is high (Tinner et al. 2013). We note that George et al. (2022) did not observe any significant trend in *A. alba* mortality over the past 25 years and that Vitasse et al. (2019) suggested that the species might thrive in a warmer climate and potentially serve as a viable replacement for more vulnerable conifer species such as *P. abies* . The significant disturbance trend observed in this study, combined with the ambiguity of previous findings, suggests that further research on the disturbance dynamics in *A. alba* is needed.

A specific pattern was observed in borers on angiosperms, particularly *Agrilus* spp. and *Scolytus* spp., which contribute to sporadic decline events of *Quercus* spp. in Europe (Thomas et al. 2002). The disturbance exhibited two clear peaks, similar to other borers, suggesting an association with the previously mentioned extreme droughts. However, contrary to bark borers on gymnosperms, the overall temporal trend for borers on angiosperms was negative. The mechanisms driving these trends are not fully understood, as borers feeding on angiosperms are understudied compared to their gymnosperm counterparts (but see, for example, Haavik et al. 2015; Macháčová et al. 2022).

Insect defoliations showed significant negative trends in the 21st century, with *L. dispar* (−3.2% per year) and *L. monacha* (−7.0% per year) being the most impactful species. The defoliation time series (Figure 2) exhibited three peaks in both insects, showing characteristic cyclic fluctuations typical for many forest Lepidoptera (Myers and Cory 2013). In the case of *L. dispar* , the last peak culminating in 2019 was considerably dampened, possibly as a result of the proliferation of the non‐native Asian entomopathogen *E. maimaiga*, which has recently affected moth populations across Europe (Georgieva et al. 2013; Zúbrik et al. 2018). The identified decrease in defoliation by *L. monacha* contradicts some previous studies indicating a possible increase in moth populations and a northward expansion under climate change (e.g., Fält‐Nardmann et al. 2018; Melin et al. 2020; Rindos et al. 2024). The decreasing disturbance trend was even more pronounced for *T. viridana* and the group of geometrid moths feeding on oaks, with annual declines of −8.9% and −15.6%, respectively. Nonetheless, the possible mechanisms behind these trends can only be hypothesized: for example, climate warming can induce collapses of cyclicity (Johnson et al. 2010), warming‐driven phenological asynchrony between larval eclosion and oak budburst can negatively impact some insect populations (Ivashov et al. 2002), and aggravating drought conditions may elevate levels of defensive compounds and reduce foliage palatability (Gely et al. 2020; Jactel et al. 2012). Still, the trends identified here need to be interpreted with caution since our data cover only a 23‐year period, and hence extend over a limited number of population cycles.

Finally, although our species‐level analyses involved important defoliators such as *L. dispar* , *L. monacha* , *T. viridana*, and the group of Geometrids, significant disturbance was also caused by other species, such as cockchafers (*Melolontha* spp.), which accounted for 13.5% of total defoliation, woodwasps (Diprionidae) on pine (8.2%), and *Dendrolimus pini* on pine (7.3%) (Figure 3). However, these species were reported from only a few countries (either due to an actual absence of significant disturbance or limitations in national survey systems), limiting the ability to analyze their Europe‐wide dynamics. Nonetheless, the high absolute levels of disturbance reported for these species underscore their importance and the need for further research.

### 4.2 Large‐Scale Disturbance Trends

Our analyses did not support the initial hypothesis of a consistent decrease in insect disturbance with latitude, as trends varied substantially across host tree species and feeding guilds (Figure 5). In contrast, we observed a strong and consistent disturbance response to the large‐scale VPD gradient—an integrative climatic variable capturing both temperature and moisture constraints (Nagavciuc et al. 2024). Contrary to expectations, this response was more pronounced in defoliators than in borers on gymnosperms (Figure 5).

The variable disturbance responses to latitude and the predominantly positive response to VPD (except for *Tomicus* spp. and *L. dispar* , Table 3) imply that regional climatic variation (e.g., due to orographic effects and the proximity of oceans, de Frenne et al. 2013) may have influenced disturbance levels more significantly than the continent‐wide latitudinal gradient. This is in agreement with Valdés‐Correcher et al. (2021), who found that climatic factors rather than latitude per se were the best predictors of insect herbivory on *Q. robur* across Europe. At the global scale, however, insect herbivory on woody plants was found to significantly increase with latitude (Liu et al. 2024), though this pattern may be substantially influenced by associational resistance linked to variation in tree species richness and other factors (Kambach et al. 2016). A noteworthy pattern was identified in *I. typographus*, the most impactful borer species in Europe, which showed a significant negative response to latitude (i.e., a northward decline in disturbance) and an insignificant response to VPD. This only partly aligns with the body of evidence on strong thermal controls over the development of the species (Baier et al. 2007) as well as the positive effect of drought on host susceptibility (Huang et al. 2020). We assume that this varied response is likely due to the strong interaction of *I. typographus* outbreaks with windthrow events (Wermelinger 2004) and the recent appearance of outbreaks initiated by extreme drought spells (Das et al. 2025; Kärvemo et al. 2023). Neither of these drivers shows a clear latitudinal pattern or aligns with continental‐scale aridity gradients represented by VPD in our analysis. Our findings thus suggest that the concept of decreasing insect impact with increasing latitude (e.g., Lim et al. 2015; Liu et al. 2024) may hold only partially true for forest insect disturbances in Europe. Instead, regional climate variability, combined with stochastic events such as droughts and windthrows that influence insect populations (Myers and Cory 2013; Potterf et al. 2025; Raffa et al. 2008; Seidl, Müller, et al. 2016), can override broader continent‐wide geographical gradients.

### 4.3 Synchrony in Disturbance Dynamics

Consistent with our initial hypothesis, we identified significant temporal correlations within insect feeding guilds but not across guilds. The highest correlations were identified for borers on *P. abies* , *P. sylvestris* , and *A. alba* (Table 5); as well as for borers on angiosperms and borers on gymnosperms (Table 4). The pattern of greater synchrony among populations of species with similar life history traits was previously also observed among defoliating insects (Raimondo et al. 2004). This correlation structure suggests the presence of shared drivers within borers, acting across insect species and/or host trees. This synchrony in disturbance is evident, for example, in the occurrence of two major peaks within all borer disturbance series (Figure 2), corresponding to the previously mentioned extreme droughts of 2003 and 2018 (Peters et al. 2020), as well as in the consistently increasing trend in borer disturbance to gymnosperms. Although analyzing the drivers of these patterns explicitly was beyond the scope of our study, growing evidence suggests that subcontinental anomalies of heat and drought are synchronizing forest disturbances across Europe (Senf and Seidl 2017, 2021) and globally (Hammond et al. 2022).

### 4.4 Data Limitations

While the data used here provide unique information about temporal trends and spatial patterns of forest insect disturbance across Europe, their use presents several challenges that need to be considered. There are no standardized protocols for data acquisition to ensure transnational comparability, survey methods may change over time in response to national priorities, and the extent of forest area covered by assessments varies between countries (see Hlásny, Perunová, et al. 2025 for a more comprehensive overview). In addition, the accuracy of disturbance attribution to specific agents depends on the training and expertise of field personnel and forest agency staff, and differs among countries. While some of these inconsistencies could be addressed through automatized gap filling procedures exploiting correlation patterns within the dataset (e.g., Patacca et al. 2023), we opted for an approach that involved iterative consultations with national experts to address issues of harmonization, completeness, and consistency (Sections 2.1 and 2.2, Appendix D). Moreover, we incorporated country as a random effect in our models, inherently addressing possible inconsistencies in survey effort, data quality, and other factors. We further mitigated uncertainty in the source data by focusing our analyses on the most impactful insect species and species groups, and on dominant host trees and tree groups. This strategy minimized the impact of potential misattribution of disturbance during field data collection, which is more likely for less common or emerging species. We conclude that the plausibility of the identified temporal trends, spatial and correlation patterns, as well as consideration of country effect in the statistical models, suggest that key uncertainties have been addressed, supporting the validity of our inferences.

### 4.5 Implications for Climate Change Adaptation

Although the accelerating disturbance by borers in gymnosperms and the declining defoliation trends in angiosperms have been reported in previous studies, these dynamics remain insufficiently addressed in forest management across Europe. Our assessment provides strong support for the need to integrate knowledge of insect disturbance dynamics into adaptation strategies and actions, rather than attempting to suppress or ignore them. Specifically, the increasing disturbance to gymnosperms should be exploited as a catalyst of forest transformation away from conifer‐oriented management, with mortality pulses opening the windows of opportunity for adaptation (Thom et al. 2017). The evidence of declining disturbance to angiosperms provides an additional incentive for their use in management, even if they have not performed as well as gymnosperms in economic terms in the past (Knoke et al. 2008). Such an approach would broadly align with adaptation priorities of the EU (Hlásny et al. 2014; Lindner et al. 2010). Importantly, the fact that these insights are derived from disturbance data already used to inform national policy and planning—and are trusted by key stakeholders—may significantly enhance their uptake compared to previous assessments. At the same time, we acknowledge considerable variation within the broad groups of angiosperms and gymnosperms, which warrants careful consideration when developing effective adaptation strategies. For example, *F. sylvatica* shows high drought sensitivity and even increased mortality following severe droughts (e.g., Obladen et al. 2021), whereas some *Quercus* species exhibit comparatively greater drought tolerance (Nosenko et al. 2025). Substantial differences also exist among gymnosperms—for example, we identified a great variation in average annual relative disturbance (Table 2), though disturbance trends were positive across all gymnosperm species. In addition, we did not account for the potential effects of invasive organisms or the roles of both native and non‐native pathogens (Jactel et al. 2020; Santini et al. 2013), which may interact with the native insect herbivores considered in this study and therefore warrant attention in the development of forest adaptation strategies.

Further, the distinct synchrony in borer disturbance across all host trees suggests that future disturbance dynamics may be manifested in recurrent pulses of tree mortality, such as those following the 2003 and 2018 droughts (Senf and Seidl 2021; Senf et al. 2020). Specifically, synchronous borer disturbance implies that disturbance fluctuations in *P. abies*—which is extensively monitored across Europe—vary in lockstep with fluctuations in less monitored species affecting *P. sylvestris* , *A. alba* , and other gymnosperms. This synchrony poses significant challenges to forest economies and timber markets, which have limited capacity to absorb such shocks (Knoke et al. 2021). Our findings thus highlight the urgency of fostering social‐ecological resilience as a core strategy for managing ecosystems under increasing biotic disturbance.

## Author Contributions

**Tomáš Hlásny:** conceptualization, methodology, visualization, writing – original draft, writing – review and editing. **Roman Modlinger:** data curation, formal analysis, methodology. **Jostein Gohli:** data curation, formal analysis, methodology. **Rupert Seidl:** conceptualization, writing – review and editing. **Paal Krokene:** conceptualization, writing – review and editing. **Iris Bernardinelli:** data curation, resources. **Simon Blaser:** data curation, resources. **Gediminas Brazaitis:** data curation, resources. **Gailenė Brazaitytė:** data curation, resources. **Eckehard G. Brockerhoff:** data curation, resources. **György Csóka:** data curation, resources. **Laura Dobor:** data curation, resources, formal analysis. **Maarten de Groot:** data curation, resources. **Mihai‐Leonard Duduman:** data curation, resources. **Massimo Faccoli:** data curation, resources. **Margarita Georgieva:** data curation, resources. **Georgi Georgiev:** data curation, resources. **Wojciech Grodzki:** data curation, resources. **Henrik Hartmann:** data curation, resources. **Anikó Hirka:** data curation, resources. **Gernot Hoch:** data curation, resources. **Tomasz Jabłoński:** data curation, resources. **Hervé Jactel:** data curation, resources. **Mats Jonsell:** data curation, resources. **Marija Kolšek:** data curation, resources. **Markus Melin:** data curation, resources. **Slobodan Milanović:** data curation, resources. **Constantin Nețoiu:** data curation, resources. **Mats Nieberg:** data curation, resources. **Bjørn Økland:** data curation, resources. **Milan Pernek:** data curation, resources. **Michaela Perunová:** data curation, resources. **Nick Schafstall:** data curation, resources. **Martin Schroeder:** data curation, resources. **Gottfried Steyrer:** data curation, resources. **Jozef Vakula:** data curation, resources. **Thomas Wohlgemuth:** data curation, resources. **Tiina Ylioja:** data curation, resources. **Andrew M. Liebhold:** conceptualization, writing – review and editing.

## Conflicts of Interest

The authors declare no conflicts of interest.

## Supporting information


**Appendix S1:** gcb70580‐sup‐0001‐Appendices.docx.

## Data Availability

The data that support the findings of this study are openly available in “Zenodo” at https://doi.org/10.5281/zenodo.15863174.
