# Supplementary material for: Divergent Trends in Insect Disturbance Across Europe's Temperate and Boreal Forests
Source: Glob Chang Biol. 2025 Oct 29;31(11):e70580. doi: 10.1111/gcb.70580 (PMC12569566; doi:10.1111/gcb.70580)

**Appendices**

**Divergent trends in insect disturbance across Europe`s temperate and boreal forests**

Running title: Insect Disturbances in European Forests

Tomáš Hlásny^1*^, Roman Modlinger^1^, Jostein Gohli^2,3^, Rupert Seidl^4,5^, Paal Krokene^2^, Iris Bernardinelli^6^, Simon Blaser^7^, Gediminas Brazaitis^8^, Gailenė Brazaitytė^8^, Eckehard G. Brockerhoff^7^, György Csóka^9^, Laura Dobor^1^, Maarten de Groot^10^, Mihai-Leonard Duduman^11^, Massimo Faccoli^12^, Margarita Georgieva^13^, Georgi Georgiev^13^, Wojciech Grodzki^14^, Henrik Hartmann^15,16,17^, Anikó Hirka^9^, Gernot Hoch^18^, Tomasz Jabłoński^14^, Hervé Jactel^19^, Mats Jonsell^20^, Marija Kolšek^21^, Markus Melin^22^, Slobodan Milanović^23,24^, Constantin Nețoiu^25^, Mats Nieberg^26,27^, Bjørn Økland^2^, Milan Pernek^28^, Michaela Perunová^1^, Nick Schafstall^1,29^, Martin Schroeder^20^, Gottfried Steyrer^18^, Jozef Vakula^30^, Thomas Wohlgemuth^7^, Tiina Ylioja^31^, Andrew Liebhold^1^

^1^Czech University of Life Sciences Prague, Faculty of Forestry and Wood Sciences, Kamýcka 129, 165 00 Prague-Suchdol, Czech Republic

^2^Norwegian Institute of Bioeconomy Research (NIBIO), 1431 Ås, Norway

^3^Norwegian Institute for Nature Research (NINA), Sognsveien 68, 0855 Oslo, Norway

^4^Technical University of Munich, TUM School of Life Sciences, Hans-Carl-von-Carlowitz-Platz 2, 85354 Freising, Germany

^5^Berchtesgaden National Park, Doktorberg 6, 83471 Berchtesgaden, Germany

^6^Regional Plant Health Service – ERSA, Friuli Venezia Giulia, Italy

^7^Swiss Federal Institute for Forest, Snow and Landscape Research WSL, Birmensdorf, Switzerland

^8^Vytautas Magnus University Agricultural Academy, Kaunas, Kaunas County, Lithuania

^9^University of Sopron, Forest Research Institute, Hegyalja str. 18, 3232 Mátrafüred, Hungary

^10^Slovenian Forestry Institute, Ljubljana, Slovenia

^11^Stefan cel Mare University of Suceava, Forestry Faculty, Universității Street 13, 720229, Suceava, Romania

^12^University of Padua, Department of Agronomy, Food, Natural Resources, Animals and the Environment, Viale dell'Università, 16 - 35020 Legnaro (PD), Italy

^13^Forest Research Institute, Bulgarian Academy of Sciences, bul. St. Kliment Ohridski 132, 1756 Sofia, Bulgaria

^14^Forest Research Institute, Sękocin Stary ul. Braci Leśnej 3, 05-090 Raszyn, Poland

^15^Institute for Forest Protection, Julius Kühn Institute (JKI) - Federal Research Centre for Cultivated Plants, Quedlinburg, Germany

^16^Faculty of Forest Sciences and Forest Ecology, Georg-August-University, Göttingen, Germany

^17^Department of Biogeochemical Processes, Max Planck Institute for Biogeochemistry, Jena, Germany

^18^Austrian Research Centre for Forests - BFW, Seckendorff-Gudent-Weg 8, 1131 Vienna, Austria

^19^INRAE, University of Bordeaux, Biogeco, 33610 Cestas, France

^20^Swedish University of Agricultural Sciences (SLU), Department of Ecology, SE-750 07 Uppsala, Sweden

^21^Slovenia Forest Service, Ljubljana, Slovenia

^22^Natural Resources Institute Finland, Yliopistokatu 6, FI-80100, Joensuu, Finland

^23^University of Belgrade, Faculty of Forestry, Belgrade, Serbia

^24^Faculty of Forestry and Wood Technology, Mendel University in Brno, Brno, Czech Republic

^25^National Institute for Research and Development in Forestry Marin “Drăcea”, Eroilor 128, 077190 Voluntari, Romania

^26^Potsdam Institute for Climate Impact Research (PIK), Member of the Leibniz Association, Telegrafenberg A 31, Potsdam, Germany

^27^European Forest Institute Bonn, Germany

^28^Croatian Forest Research Institute, Cvjetno naselje 41, 10450 Jastrebarsko, Croatia

^29^Nature Research Center, Akademijos St. 2, 08412 Vilnius, Lithuania

^30^National Forest Centre, Department of Forest Protection and Forest Protection Service, Lesnícka 11, 969 01 Banská Štiavnica, Slovakia

^31^Natural Resources Institute Finland, Latokartanonkaari 9, FI-00790, Helsinki, Finland

*Corresponding author: Tomáš Hlásny, email: [hlasny@fld.czu.cz](mailto:hlasny@fld.czu.cz), phone: +421 905 708 539

**Citation:**

Hlásny, T., Modlinger, R., Gohli, J., Seidl, R., Krokene, P., Bernardinelli, I., Blaser, S., Brazaitis, G., Brazaitytė, G., Brockerhoff, E. G., Csóka, G., de Groot, M., Duduman, M.-L., Faccoli, M., Georgieva, M., Georgiev, G., Grodzki, W., Hartmann, H., Hirka, A., Hoch, G., Jabłoński, T., Jactel, H., Jonsell, M., Kolšek, M., Melin, M., Milanović, S., Nețoiu, C., Nieberg, M., Økland, B., Pernek, M., Perunová, M., Schafstall, N., Schroeder, M., Steyrer, G., Vakula, J., Wohlgemuth, T., Ylioja, T., and Liebhold, A. (2025). Divergent trends in insect disturbance across Europe’s temperate and boreal forests. *Global Change Biology*. https://doi.org/10.1111/gcb.70580.

**Appendix A: Data overview**

Table A1 Description of the compiled data on forest damage by insect herbivores in Europe. NUTS2: Nomenclature of Territorial Units for Statistics.

| **Country** | **Spatial resolution*** | **Years covered** | **Reported pest species** |
| --- | --- | --- | --- |
| Austria (AT) | 9 NUTS2 regions | 2000-2022 | Bark borers on pine, *Tortrix viridana, Erannis defoliaria, Operophtera brumata, Operophtera fagata, Diprion sp., Gilpinia* sp*. Neodiprion sertifer, Ips typographus, Phaenops cyanea, Pityokteines spp., Cryphalus spp., Pissodes* spp*., Tetropium gabrieli, Tomicus* spp*., Lymantria dispar, Pityogenes chalcographus* (17 species or species groups) |
| Bulgaria (BG) | Country | 2005-2022 | *Ips acuminatus, Ips sexdentatus, Ips typographus, Neodiprion sertifer, Thaumetopoea pityocampa, Erannis defoliaria, Operophtera brumata, Operophtera fagata, Lymantria dispar, Tortrix viridana* (10 species) |
| Croatia (HR) | Country | 2000-2022 | Bark borers on pine, *Ips typographus, Pityokteines* spp*., Cryphalus spp., Pissodes* spp*., Thaumetopoea pityocampa, Erannis defoliaria, Operophtera brumata; Operophtera fagata, Lymantria dispar* (10 species or species groups) |
| Czechia (CZ) | 13 NUTS2 regions | 2000-2022 | Bark borers on spruce, Bark borers on larch, *Cephalcia abietis, Tortrix viridana, Erannis defoliaria, Operophtera brumata, Operophtera fagata, Dendroctonus micans, Ips acuminatus, Ips duplicatus, Ips sexdentatus, Neodiprion sertifer, Panolis flammea, Phaenops cyanea, Pityokteines* spp*., Cryphalus* spp*., Pissodes* spp*.,* *Polygraphus poligraphus, Pristiphora abietina, Scolytus intricatus, Scolytus ratzeburgii, Tetropium gabrieli, Tomicus* spp*., Zeiraphera griseana, Lymantria dispar, Lymantria monacha* (26 species or species groups) |
| Germany (DE) | 15 NUTS2 regions | 2000-2022 | Bark borers on pine, Bark borers on spruce, Bark borers on pine, Bark borers on larch, *Agrilus* spp, *Bupalus piniaria, Erannis defoliaria, Operophtera brumata, Operophtera fagata, Dendroctonus micans, Dendrolimus pini, Diprion* spp*., Gilpinia* spp*. Neodiprion sertifer, Erannis defoliaria, Ips acuminatus, Ips amitinus, Ips sexdentatus, Ips typographus, Melolontha* spp*., Neodiprion sertifer, Operophtera* spp*., Panolis flammea, Phaenops cyanea, Pissodes pini, Pityokteines* spp*., Cryphalus* spp*., Pissodes* spp*., Pristiphora abietina, Scolytus intricatus, Scolytus ratzeburgii, Scolytus scolytus, Tetropium gabrieli, Tetropium* spp*., Thaumetopoea pityocampa, Thaumetopoea processionea, Tomicus spp., Wood wasps, Zeiraphera griseana, Pityogenes bidentatus, Ips cembrae, Saperda carcharias, Agrilus* spp*, Trypodendron lineatum, Trypodendron domesticum, Trypodendron signatum, Xyleborus monographus, Erannis defoliaria; Lymantria dispar, Lymantria monacha, Pityogenes chalcographus, Tortrix viridana* (55 species or species groups) |
| Finland (FI) | 17 NUTS2 regions | 2012-2022 | *Ips typographus* (1 species) |
| Hungary (HU) | Country | 2000-2022 | *Ips typographus, Lymantria dispar* (2 species) |
| Italy (IT) | 1 NUTS, Friuli-Venezia Giulia | 2000-2022 | *Epinotia tedella, Hylesinus fraxini, Ips acuminatus, Ips sexdentatus, Ips typographus, Pissodes piceae, Pityogenes chalcographus, Xyleborus dispar* (8 species) |
| Lithuania (LT) | Country | 2000-2022 | *Dendroctonus micans, Dendrolimus pini, Diprion* spp*., Gilpinia sp. Neodiprion sertifer, Ips acuminatus, Ips typographus, Panolis flammea, Phaenops cyanea, Pissodes pini, Polygraphus poligraphus, Scolytus ratzeburgii, Scolytus scolytus, Tomicus* spp*., Erannis defoliaria; Operophtera brumata; Operophtera fagata, Lymantria dispar, Lymantria monacha, Pityogenes chalcographus, Tortrix viridana* (22 species) |
| Poland (PL) | 17 NUTS2 regions* | 2003-2022 | *Agrilus* spp*, Bupalus piniaria, Dendrolimus pini, Diprion* spp*., Gilpinia* spp*. Neodiprion sertifer, Ips acuminatus, Ips typographus, Melolontha spp., Panolis flammea, Phaenops cyanea, Tomicus* spp*., Erannis defoliaria; Operophtera brumata; Operophtera fagata, Lymantria monacha, Tortrix viridana* (17 species) |
| Romania (RO) | Country | 2000-2022 | *Ips duplicatus, Ips typographus, Clostera anastomosis, Erannis defoliaria; Operophtera brumata; Operophtera fagata, Lymantria dispar, Tortrix viridana* (8 species) |
| Serbia (RS) | Country | 2000-2022 | *Ips typographus, Lymantria dispar* (2 species) |
| Slovakia (SK) | Country | 2000-2022 | *Ips acuminatus, Ips duplicatus, Ips typographus, Lymantria dispar, Pityogenes chalcographus* (5 species) |
| Slovenia (Sl) | Country | 2000-2022 | Bark borers on pine, *Ips typographus, Pityokteines* spp*., Cryphalus* spp*., Pissodes* spp*., Pityogenes chalcographus* (6 species or species groups) |
| Sweden (SE) | Country | 2006-2022 | *Ips typographus* (1 species) |
| Switzerland (CH) | Country | 2000-2022 | *Ips typographus* (1 species) |

*in Poland, the data were recalculated from national forestry districts to the NUTS2 regions for consistency

**Appendix B: National data acquisition, pre-processing and reporting procedures**

| Country | Data origin, processing and quality |
| --- | --- |
| Austria (AT) | In the Austrian Documentation of Forest Damaging Factors, data are recorded based on estimates for affected area and volume by foresters of the district forest authorities in their supervised district. The foresters are trained to estimate volume and areas; volume estimates are often controlled by comparison with logging records from forest enterprises in the district. Data are entered into a database provided by Austrian Research Centre for Forests (BFW, <https://www.bfw.gv.at/en/).> After a first check at district level, collected data are reviewed by the forest protection specialist at federal state level. Questionable entries are sent back to the district forester for confirmation or revision. After approval at state level, the data are compiled by BFW. In cases where a damaging agent cannot be determined by the forester, experts at BFW or the state forest protection specialist are consulted. The whole forest area in Austria is covered by this documentation. |
| Bulgaria (BG) | The data used were obtained from the Information System of the Executive Forest Agency of Bulgaria. Damage caused by abiotic and biotic factors, including bark beetles and other xylophagous insect pests, is reported by responsible foresters for state forest units (State Forestry Enterprises and State Hunting Enterprises), and by licensed foresters for private and municipal forests, as well as forests within National and Natural Parks. Specialists from the Regional Forest Directorates evaluate each initial record of abiotic damage entered the Information System, while biotic damage caused by xylophagous and other insect pests is assessed by specialists from the Forest Protection Stations (there are three in Bulgaria—Sofia, Plovdiv, and Varna). The area of the affected stands, along with the volume of attacked and cut stems, is entered into the central database by the responsible specialists. Information collected in the field is obligatorily verified and coordinated between forest owners and the responsible institutions. Decisions regarding sanitary felling are made by commissions that include representatives from the regional Forest Protection Station. In complex or difficult-to-identify cases, experts from the Forest Research Institute and the University of Forestry in Sofia are consulted to determine the causative agents. The Forest Protection Stations prepare an annual report on the anticipated rate of damage caused by insect pests and diseases in forests, which is submitted to the Executive Forest Agency. |
| Croatia (HR) | The data for Croatia were taken from the database related to reporting and forecasting activities defined in the Plant Health Act, where the Croatian Forest Research Institute is designated as the institution authorized to collect and interpret biotic and abiotic factors causing damage in forests. The data have been systematically collected and maintained since 1980, and digitally since 2008 at the Croatian Forest Research Institute. According to the Plant Health Act, all forest owners are obliged to report forest damage and send the data to the Croatian Forest Research Institute. In the field, data are collected by experts—forestry engineers—who send them to the forest administrations, which then forward the data to the Institute. At the Institute, the data are evaluated by highly qualified experts, including PhD-level scientists, and, when necessary, verified in the field. In cases of new types of damage, Institute experts may be dispatched to the field, and the results of their analyses are incorporated into the database. There is an annual reporting obligation, and the report is submitted to the Ministry of Agriculture and Forestry. |
| Czechia (CZ) | Damage caused by borers is reported on a monthly basis by professional foresters, who are trained to estimate the volume of damaged and harvested wood and to attribute the damage to the primary pest species. When unusual damage patterns are observed (for example, based on distinctive galleries under the bark), specialists from the national forest protection service are consulted to identify the causal agent, either in the field or through laboratory analysis of collected samples. Once field data are submitted to the central database, they undergo a consistency check. Any anomalies in reported values, such as unexpected volumes or damage causes, are reviewed in consultation with forest owners and revised as necessary. A similar procedure is followed for defoliators. Affected areas are typically classified into three defoliation severity classes. In all larger events, experts are called upon to determine the causative agent. |
| Finland (FI) | Salvage logging data in Finland is compiled by the Finnish Forest Centre and covers only private forests, which represent approximately 74% of the total forest area. However, there is no standardized protocol for data collection, and the expertise of the individuals reporting the data can vary considerably. The dataset provides information on the number of hectares harvested due to damage caused by spruce bark beetles. Since 2014, it has been mandatory to indicate whether logging was carried out as a result of insect damage. |
| Germany (DE) | Germany is federal country and forest damage assessment is carried out by the federal states. States have different reporting routines and cycles, and not all damaging agents are necessarily present in all states. Within the federal states, damage assessment is done by professional foresters in their respective districts, and reported to the state agency responsible for curing and collecting the data. Twice a year, state agencies report cumulated damage data to the responsible federal ministry, and this reporting is the basis for publication in the forestry magazine. Symptoms of causal damage agents are identified in the field (e.g., frass surrounding the base of the trunk of a tree, presence and structure of galleries under the bark) and reported along with metrics of amount of damage, either in hectares (for extensive damage) or volume of commercial wood affected (for diffuse damage). Criteria used for identification of specific damaging agents are homogenized across state agencies. In case of ambiguous identification of damaging agents, district foresters sent samples to the state agency for close examination. Potential erroneous reporting (inconsistent values, uncertainty in damage agent identification) is reviewed by the state agency and is then reevaluated after consultation with the forest owner. |
| Hungary (HU) | Between 2000 and 2011 forest owners and managers managing forests more than 200 hectares were obliged to report both abiotic and biotic damage types from their area. These data were summarized by our department in the FRI. These data represented ca. 3/4 of the total forested area. Since 2012 the reports represent almost the total forested area. These data are collected by the Forest Inventory Department of the Ministry of Agriculture but analysed together with our department. The bark beetle damage refers almost exclusively for *I. typographus* and to a minor extent to other bark beetles of conifers. |
| Italy (IT) | Data on forest pest infestations were obtained from the phytopathological forest inventory of the Friuli Venezia Giulia region (called BAUSINVE). Since 1994 BAUSINVE provides information about forest pests and diseases occurring in the regional forests. Forest health of the whole region is monitored daily via ground-based survey by about 60 foresters working for the Regional Forest Service and supervised by a team of entomologists and pathologists from scientific institutes. When an outbreak occurs, foresters compile specific reports, including information concerning both the infesting pest (species, development instar, population density) and the total damage (number of trees attacked, volume of infested timber, defoliation area). During the survey, the foresters also classify the characteristics of the attacked forests, collecting data on elevation, tree density, composition, structure, type of forest and management. Data are then recorded and stored in an electronic database. Our database thus derives from a long-term intensive forest health monitoring covering a 30-year period (1994–2024). Each recorded infestation spot composed of at least 5 infested trees was visited to identify the biotic agent causing tree mortality, recording the number of killed trees, the volume of timber loss (m3) and the site characteristics. Using these data, we built a time series of annual timber loss for the whole region by summing the volume (m3) of trees killed in all the spots occurring each year in each forest type. |
| Lithuania (LT) | Data for Lithuania is taken from annual "Review of Sanitary conditions" issued by the State Forest Service under the Ministry of Environment. The material contains comprehensive overview about biotic and abiotic damages in the state-owned forests. The data about insects, diseases, wildlife, wind, snow, fire, and other damages are provided for regional districts. The data is based on annual reports from forest regional districts and commonly include both damage and applied measures, such as sanitary cuttings report. Damage is reported as volume (m³) and area (ha), based primarily on visual assessments by forestry professionals. Damaged areas ≥ 1 ha are monitored by State Forest Service forest-health (phytosanitary) specialists. Applied measures are reported as felled volume and treated area and are tracked using instrument-based measurements. |
| Poland (PL) | Damage caused by abiotic and biotic agents is collected by the professional staff of the State Forests (managing almost 80% of forests in Poland), including specialized staff of the Forest Protection Service, dedicated for supervision and advisory activities, The data is stored in the State Forests IT System (SILP). SILP is an information technology tool dealing with complex economic processes and their integrated approach, coherently showing the state of management at all levels: forestry unit, forest district, SF regional directorate, SF General-Directorate. Data on the occurrence of forest defoliating insects and the risk of damage, as well as on the protective treatments, are introduced into the SILP and stored for current and future use. Data concerning the volume of processed trees damaged by the abiotic agents and/or bark beetles is collected based on the measurement and recording of timber (with a special code indicating the damage) after felling but before selling. The data is then aggregated on higher levels (from forest subcompartment to the General Directorate of SF) and used for the assessment and forecast of health status and threats to the forests, presented in yearly published reports. |
| Romania (RO) | Forest health issues are reported monthly by forest protection officers at the level of each forest management unit. These reports include data such as the area of forest affected, the volume of timber impacted, and the pests or pest groups involved. When damage from a particular pest increases or new pests emerge, forest managers consult with forest protection specialists from INCDS or the Faculties of Forestry–either in the field or in specialized laboratories–to ensure accurate identification and assessment of the damage. Centralized reporting at the national level is carried out only for forests managed by the National Forest Administration, which represents approximately 50% of Romania’s forest area. Any abnormal values observed in the reports are verified in the field by specialists and corrected if necessary. The data used in this study were provided by the Forest Protection Service of the National Forest Administration, via the National Forestry Research-Development Institute (INCDS) "Marin Drăcea". |
| Serbia (RS) | In Serbia, the Forest Protection Service is supported by the Directorate of Forests under the Ministry of Agriculture, Forestry, and Water Management. The service is being provided by two Institutes of Forestry under five-year contracts. State forest enterprises and national parks collect data on the presence of pests and diseases, as well as forest damage levels, and submit this information to the institutes for verification and aggregation. Periodically, forest protection experts from the institutes conduct random field inspections to validate the reported data. Both institutes are equipped with entomological and phytopathological laboratories for further evaluation when needed. In cases of extreme events, such as insect or disease outbreaks, special teams are formed to assess damage levels, recommend mitigation measures, and coordinate control efforts. The collected data are submitted to the Directorate of Forests through semi-annual reports, which are subsequently forwarded to the Statistical Office of the Republic of Serbia. The data presented in this paper were obtained from the Statistical Office of the Republic of Serbia. |
| Slovakia (SK) | Damage caused by bark and wood boring beetles is reported to National Forest Centre by professional foresters on a yearly basis, who are trained to estimate the volume of damaged and harvested wood and to attribute the damage to the primary pest species. When unusual damage patterns are observed (for example, based on distinctive galleries under the bark), specialists from the national forest protection service are consulted to identify the causal agent, either in the field or through laboratory analysis of collected samples. Once field data are submitted to the central database, they undergo a consistency check. Any anomalies in reported values, such as unexpected volumes or damage causes, are reviewed in consultation with forest owners and revised as necessary. A similar procedure is followed for defoliators. Affected areas are typically classified into three defoliation severity classes. In all larger events, experts are called upon to determine the causative agent. |
| Slovenia (Sl) | Data on sanitation felling of trees affected by insects and diseases is collected by the Slovenian Forest Service on a nationwide scale. For each felled tree, the causal agent is identified, and the tree volume is measured. The data is collected at the department level by local foresters from the SFS and entered into the central database. In case of questions, the forester who entered the data on insect- or disease-related damage is consulted. If the damaging agent cannot be determined by the forester, experts from the Slovenian Forestry Institute are consulted. |
| Sweden (SE) | Volumes of Norway spruce killed by the spruce bark beetle (*Ips typographus*) were only estimated during outbreaks. The method used have differed somewhat between outbreaks. The two most used method have been: 1) Inventory of sample plots of the Swedish National Forest Inventory for trees killed by I. typographus in the current year. Also already cut killed trees were included (identified by presence of fallen bark with I. typographus galleries and the freshness of the stump). 2) Inventory of reference forest properties. In both cases the inventories were conducted in the autumn and then scaled up for each region. |
| Switzerland (CH) | The volume (m^3^) of spruce infested by *Ips typographus* is reported from each forest district of Switzerland (N=673) in an annual survey. The data include the volume of infested spruce harvested in sanitary fellings and the volume of infested spruce left standing in forests. The collection, quality check and maintenance of the data is ensured by the Forest Protection group at the Swiss Federal Institute for Forest, Snow and Landscape Research WSL. |

**Appendix C:** **Data preprocessing**

Table C1 Modifications made to the forest damage data reported by different countries in order to harmonize the dataset and facilitate statistical analyses. Two types of correction are indicated (1) upscaling from the area covered by data (public forest land) to the entire forest area in each country; and (2) recalculation of damage values reported in hectares to cubic meters (see Appendix C). In the latter case, for example, the value of 157.5 indicates that a given insect affected, on average 157.5 m^3^, of wood per hectare. Data from the remaining seven countries included in the study (see Appendix A) were used as reported.

| **Country** | **Modification of the source data** |
| --- | --- |
| Bulgaria | (2) Recalculating ha to m^3^ by using a factor of 157.5 for ‘bark borers on spruce’ and 55 ‘for bark borers on pine’ |
| Czechia | (1) Upscaling all values by a factor of 1.42 (only 70% of the forest is covered by damage assessment). |
| Germany | (Coefficients for individual federal states of Germany are stated in separate table B2) |
| Finland | (2) Recalculating ha to m^3^ by using a factor of 122 for ‘bark borers on spruce. |
| Hungary | (2) Recalculating ha to m^3^ by using a factor of 160 for ‘bark borers on spruce’ |
| Lithuania | (1) Upscaling all values by a factor of 2 (only 50% of the forest is covered by damage assessment). |
| Poland | (1) Upscaling all values by a factor of 1.25 (only 80% of the forest was covered by damage assessment). The collectively reported category ‘bark borers on pine’ was split to species-specific categories: *Phaenops cyanea, Tomicus* spp. and *Ips acuminatus* in the proportions 0.7, 0.1 and 0.2, respectively. However, the proportions differed between NUTS regions and over time, as assessed by expert estimates. |
| Romania | (1) Upscaling all values by a factor of 1.56 (only 64% of the forest, on average, is covered by damage assessment).  (2) Recalculating ha to m^3^ by using a factor of 1.809 for ‘bark borers on spruce’. |

Table C2 Modifications to German forest-damage data to harmonize the dataset and enable statistical analysis: (1) upscaling from the reporting domain (public forest) to the total forest area; and (2) converting damage reported in hectares to volume (m³). NUTS2: Nomenclature of Territorial Units for Statistics.

| **State (NUTS code)** | Proportion of forest land covered by data / upscaling coefficient | Species-specific coefficients used for recalculating ha to m^3^ (coefficients were based on cases where damage was reported in both units) |
| --- | --- | --- |

| Baden-Württemberg | (DE1) | 34% / 2.94 | *Ips typographus:* 54 |
| --- | --- | --- | --- |
| Bavaria | (DE2) | 30% / 3.33 | *Pityogenes chalcographus:* 17 |
| Berlin | (DE3) | 80% / 1.25 | *Tetropium* spp.: 3 |
| Brandenburg | (DE4) | 34% / 2.94 | Bark borers on pine: 4.9 |
| Bremen | (DE5) | 98% / 1.02 | Bark borers on fir: 166 |
| Hamburg | (DE6) | 70% / 1.43 | Bark borers on larch: 16.4 |
| Hessen | (DE7) | 42% / 2.38 | Bark borers on deciduous: 36 |
| Mecklenburg-Vorpommern | (DE8) | 50% / 2 |  |
| Lower Saxony | (DE9) | 33% / 3.03 |  |
| North Rhine-Westphalia | (DEA) | 17% / 5.88 |  |
| Rhineland-Palatinate | (DEB) | 12% / 8.33 |  |
| Saarland | (DEC) | 30% / 3.33 |  |
| Saxony | (DED) | 30% / 3.33 |  |
| Saxony-Anhalt | (DEE) | 30% / 3.33 |  |
| Schleswig-Holstein | (DEF) | 20% / 5 |  |
| Thuringia | (DEG) | 40% / 2.5 |  |

**Appendix D Amount of main host species within the study area**

Table D1 Amounts of host tree in the participating countries.

| **Country** | Forest cover | | Norway spruce | | Scots pine | | Oaks | | Fir | | Gymnosperms | | Angiosperms | |
| --- | --- | --- | --- | --- | --- | --- | --- | --- | --- | --- | --- | --- | --- | --- |
|  | [th. ha] | [%] | [th. ha] | [mil m^3^] | [th. ha] | [mil. m^3^] | [th. ha] | [mil. m^3^] | [th. ha] | [mil. m^3^] | [th. ha] | [mil. m^3^] | [th. ha] | [mil. m^3^] |
| Austria (AT) | 3 889 | 46.4 | 1 651 | 726 | 181 | 89 | 67 | 32 | 91 | 53 | 2 106 | 957 | 895 | 254 |
| Bulgaria (BG) | 3 900 | 35.1 | 134 | 0 | 535 | 0 | 166 | 0 | - | - | 1 706 | 287 | 2 193 | 369 |
| Czech Republic (CZ) | 2 680 | 33.9 | 1 285 | 399 | 426 | 103 | 182 | 35 | 27 | 8 | 1 865 | 543 | 693 | 125 |
| Germany (DE) | 11 468 | 32.1 | 2 763 | 1 180 | 2 428 | 757 | 1 127 | 343 | 183 | 89 | 5 353 | 2 036 | 4 718 | 1 338 |
| Finland (FI) | 22 409 | 66.2 | 3 577 | 652 | 8 259 | 966 | - | - | - | - | 11 836 | 1 618 | 1 081 | 418 |
| Croatia (HR) | 1 940 | 34.3 | 80 | 13 | 84 | 19 | 558 | 125 | 223 | 34 | 387 | 66 | 1 699 | 359 |
| Hungary (HU) | 2 053 | 22.1 | 12 | 5 | 108 | 34 | 510 | 159 | - | - | 314 | 55 | 1 739 | 305 |
| Switzerland (CH) | 1 221 | 30.9 | 462 | 181 | 41 | 12 | 24 | 9 | 136 | 64 | 744 | 287 | 414 | 134 |
| Italy (IT) | 420 | 42 | 25 | 3 | 43 | 5 | 36 | 4 | 19 | 2 | 126 | 14 | 294 | 32 |
| Lithuania (LT) | 2 160 | 34.5 | 450 | 0 | 706 | 0 | 34 | 0 | - | - | 1 259 | 274 | 901 | 196 |
| Poland (PL) | 9 464 | 30.3 | 365 | 105 | 4 911 | 1 251 | 546 | 124 | 153 | 52 | 5 430 | 1 408 | 1 647 | 386 |
| Romania (RO) | 6 982 | 29.3 | 1 388 | 515 | 62 | 23 | 1 127 | 314 | 295 | 152 | 1 777 | 753 | 5 152 | 1 602 |
| Serbia (RS) | 3 050 | 31 | 86 | 19 | 126 | 16 | 721 | 82 | 26 | 10 | 243 | 46 | 2 009 | 295 |
| Sweden (SE) | 27 980 | 62.5 | 11 806 | 1 365 | 11 240 | 1 300 | 329 | 38 | - | - | 23 378 | 2 703 | 4 596 | 532 |
| Slovenia (SI) | 1 185 | 58.5 | 799 | 104 | 256 | 20 | 576 | 31 | 307 | 27 | 534 | 187 | 651 | 228 |
| Slovakia (SK) | 1 951 | 39.8 | 439 | 129 | 129 | 32 | 253 | 57 | 78 | 25 | 716 | 198 | 1 226 | 283 |
| Total | 112 817 | – | 25 322 | 5 396 | 29 534 | 4 626 | 6 255 | 1 352 | 1 538 | 516 | 57 774 | 11 432 | 29 907 | 6 855 |

Table D2 Sources of data on the amount of host tree species in the participating countries

| **Country** | **Source** |
| --- | --- |
| Austria (AT) | Österreichische Waldinventur (Austrian National Forest Inventory, ÖWI 2018-23); BFW-Bundesforschungszentrum für Wald (BFW-Austian Research Centre for Forests). https://www.waldinventur.at/#/en |
| Bulgaria (BG) | Bulgarian Forest Executive Agency, 2005-2022, <https://www.iag.bg/> |
| Czech Republic (CZ) | Forest Management Institute, Forest Management Plan Data 2018-2023 |
| Germany (DE) | Thünen-Institut, 2012. Dritte Bundeswaldinventur - Ergebnisdatenbank [Third national forest inventory]. https://bwi.info, accessed 23.04.2025. |
| Finland (FI) | 11th and 12th National Forest Inventory of Finland, 2009 - 2018. https://statdb.luke.fi/PxWeb/pxweb/en/LUKE/LUKE__04%20Metsa__06%20Metsavarat/1.12_Puulajien_vallitsevuus_ja_metsikoiden.px/ |
| Croatia (HR) | Čavlović, J. 2010. The First National Forest Inventory of the Republic of Croatia. Ministry of Regional Development, Forestry and Water Management of the Republic of Croatia. Zagreb: . pp 296 |
| Hungary (HU) | Division of Forest Planning, Hungarian Ministry of Agriculture, Forest Inventory 2015-2019 |
| Switzerland (CH) | National Forest Inventory NFI 2009-2017: Brändli, U.-B.; Abegg, M.; Allgaier Leuch, B. (Red.) 2020:Schweizerisches Landesforstinventar. Ergebnisse der vierten Erhebung 2009–2017. Birmensdorf, Eidgenössische Forschungsanstalt für Wald, Schnee und Landschaft WSL. Bern, Bundesamt für Umwelt. 341 S. |
| Italy (IT) | Schelhaas, M.J., Varis, S., Schuck, A. and Nabuurs, G.J., 2006, EFISCEN Inventory Database, European Forest Institute, Joensuu, Finland, http://www.efi.int/portal/virtual_library/databases/efiscen/ |
| Lithuania (LT) | Schelhaas, M.J., Varis, S., Schuck, A. and Nabuurs, G.J., 2006, EFISCEN Inventory Database, European Forest Institute, Joensuu, Finland, http://www.efi.int/portal/virtual_library/databases/efiscen/ |
| Poland (PL) | Forest Data Bank (BDL) (https://www.bdl.lasy.gov.pl/portal/) |
| Romania (RO) | Marin Ghe., Bouriaud O., Calotă I.C., Nițu D.M., Dumitru M., 2019: Inventarul Forestier Național din România. Ciclul I (2008-2012). Editura Silvică, Voluntari Romania, 171 p. (htps://roifn.ro/pdfs/Inventarul_forestier_national_din_Romania_Ciclul_I_2008-2012.pdf) |
| Serbia (RS) | THE NATIONAL FOREST INVENTORY OF THE REPUBLIC OF SERBIA - The growing stock of the Republic of Serbia from 2009. https://upravazasume.gov.rs/wp-content/uploads/2015/12/The-national-forest-inventory-of-the-Republic-of-Serbia-1.pdf |
| Sweden (SE) | Swedish Statistical Yearbook of Forestry 2014, Swedish Forest Agency 2014 |
| Slovenia (SI) | Slovenian Forest Service, period from 2015 till 2024. |
| Slovakia (SK) | National Forest Centre - Forestry data (https://gis.nlcsk.org/IBULH/LesHospSI/LesHospSI) |

**Appendix E Relationship between the mean annual air temperature and vapor pressure deficit**

**
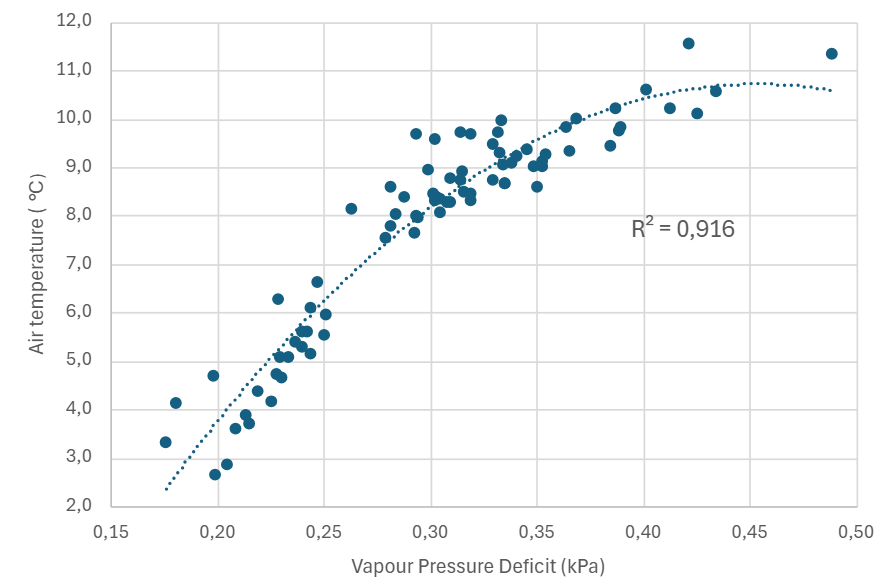
**

Fig. E1 Relationship between the mean annual air temperature and vapor pressure deficit across Europe. Average values for the period 2000-2022 are presented. Each point represents a country or administrative district (N=82) for which data on forest insect disturbance were available. The values were extracted for the forested areas within each spatial entity.

**Appendix F: Individual linear models fitted to data on forest disturbance cause by different insect species and species groups**

N – number of values (time series × number of years with data); Response variable: Forest disturbance level (hectares for defoliators and cubic meters for borers). Predictor variables: Year, Latitude, Longitude, Vapour Pressure Deficit, Host proportion (logarithm).


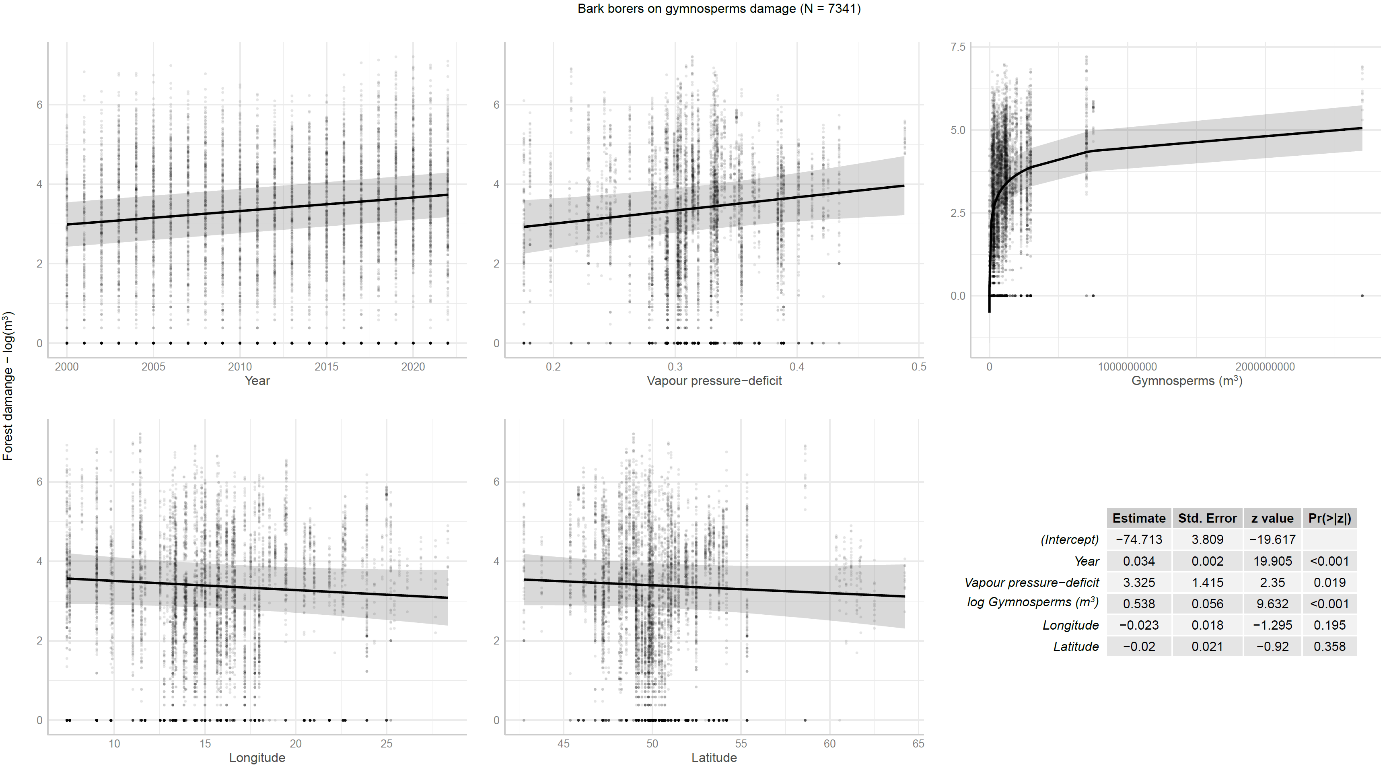


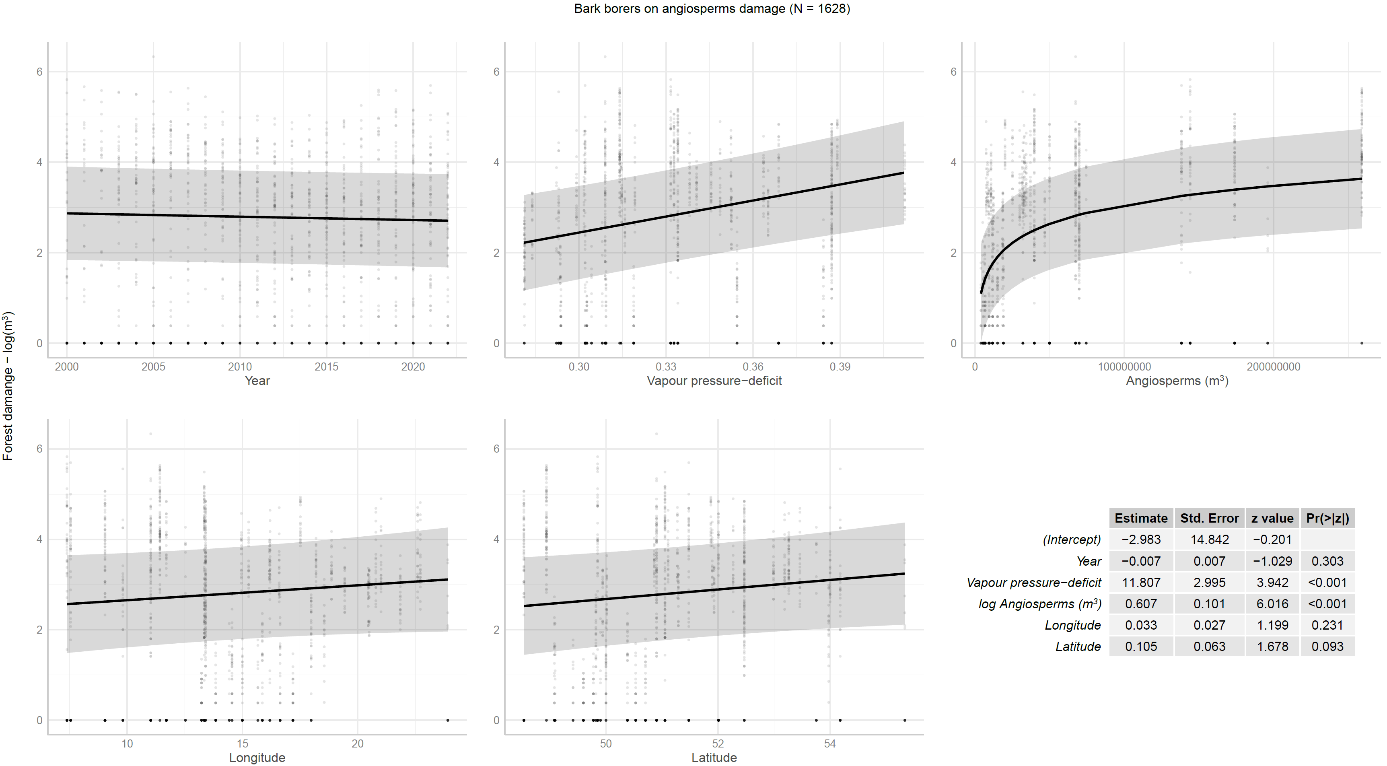


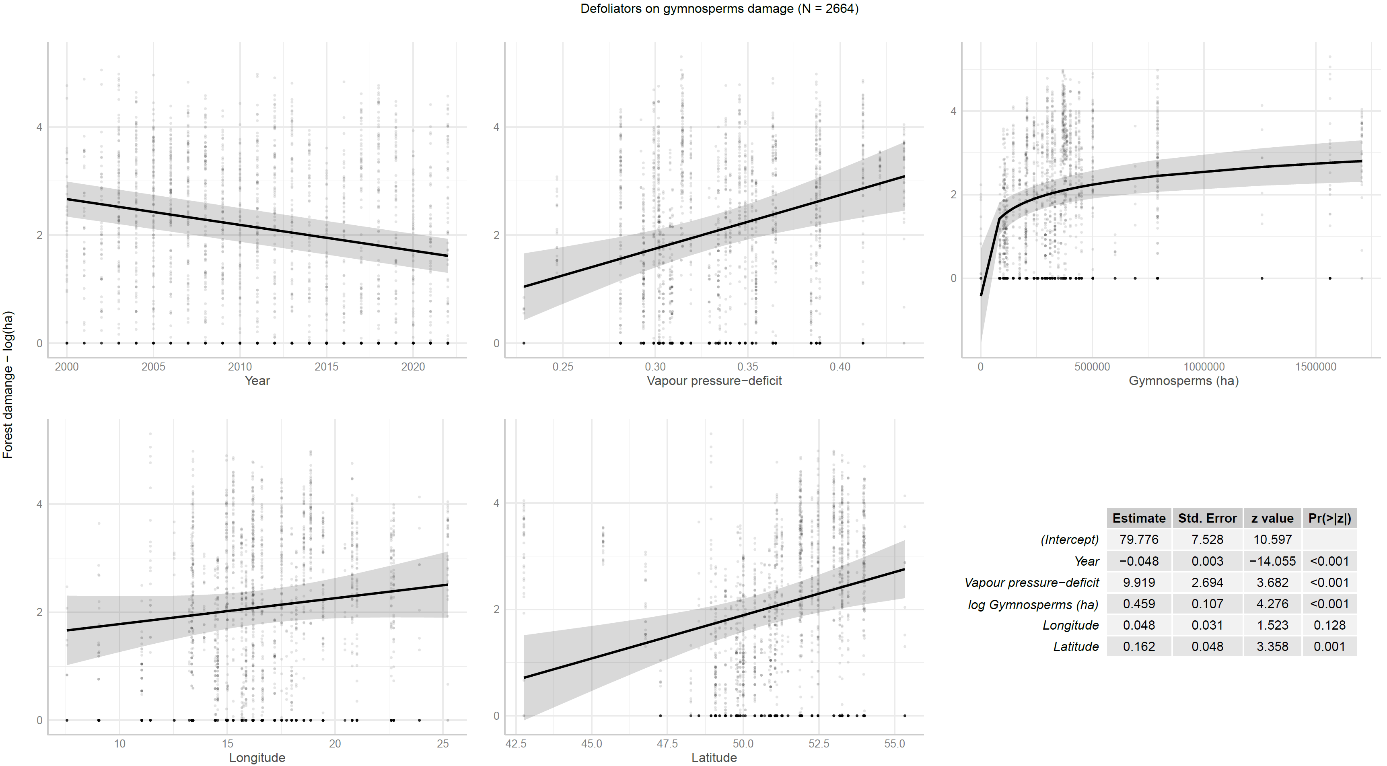


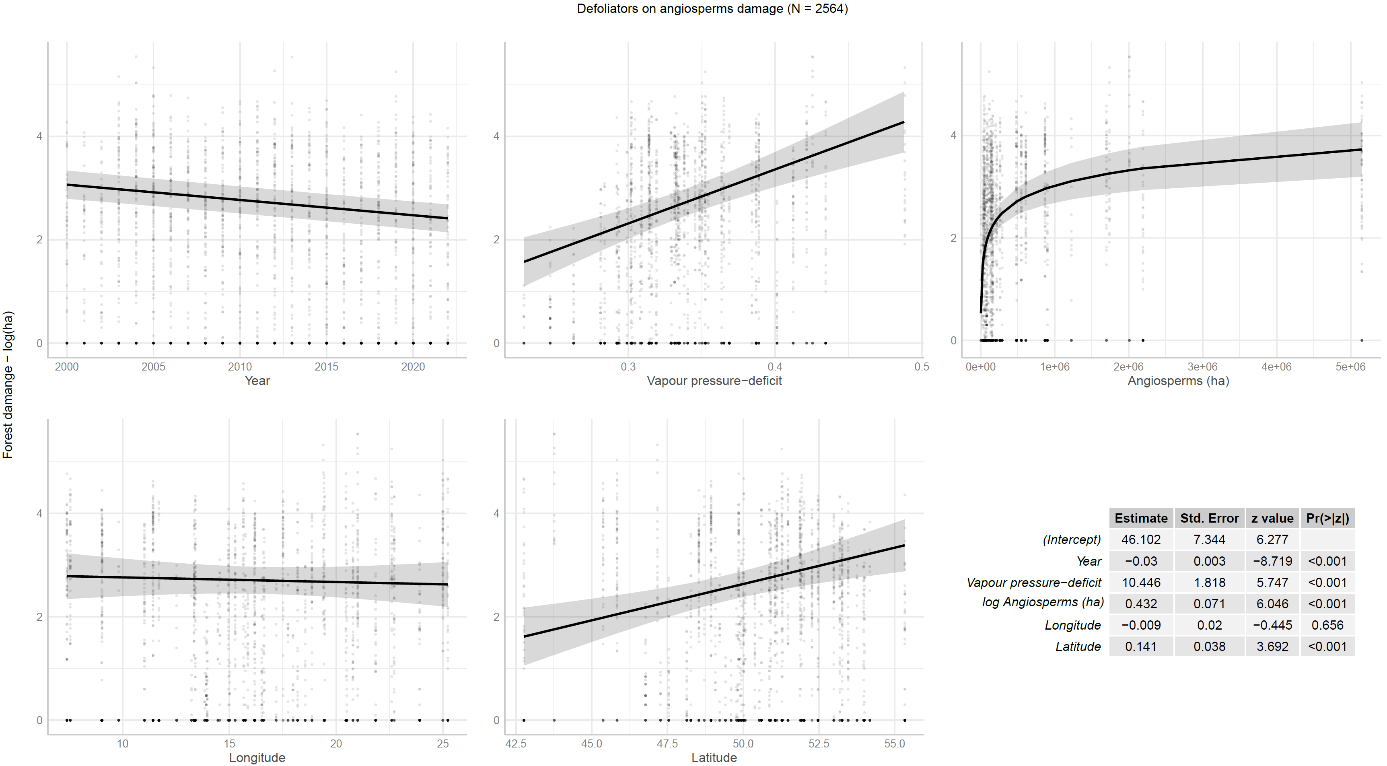


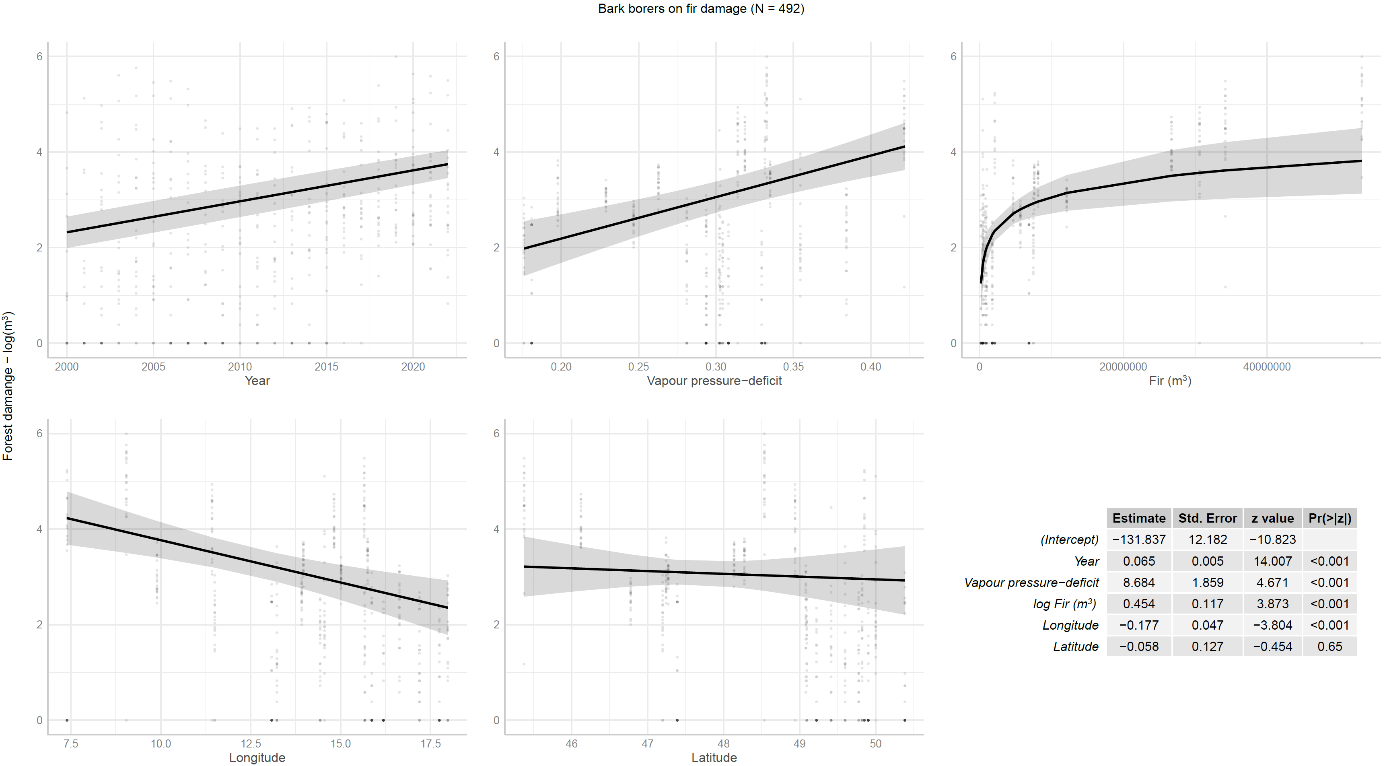


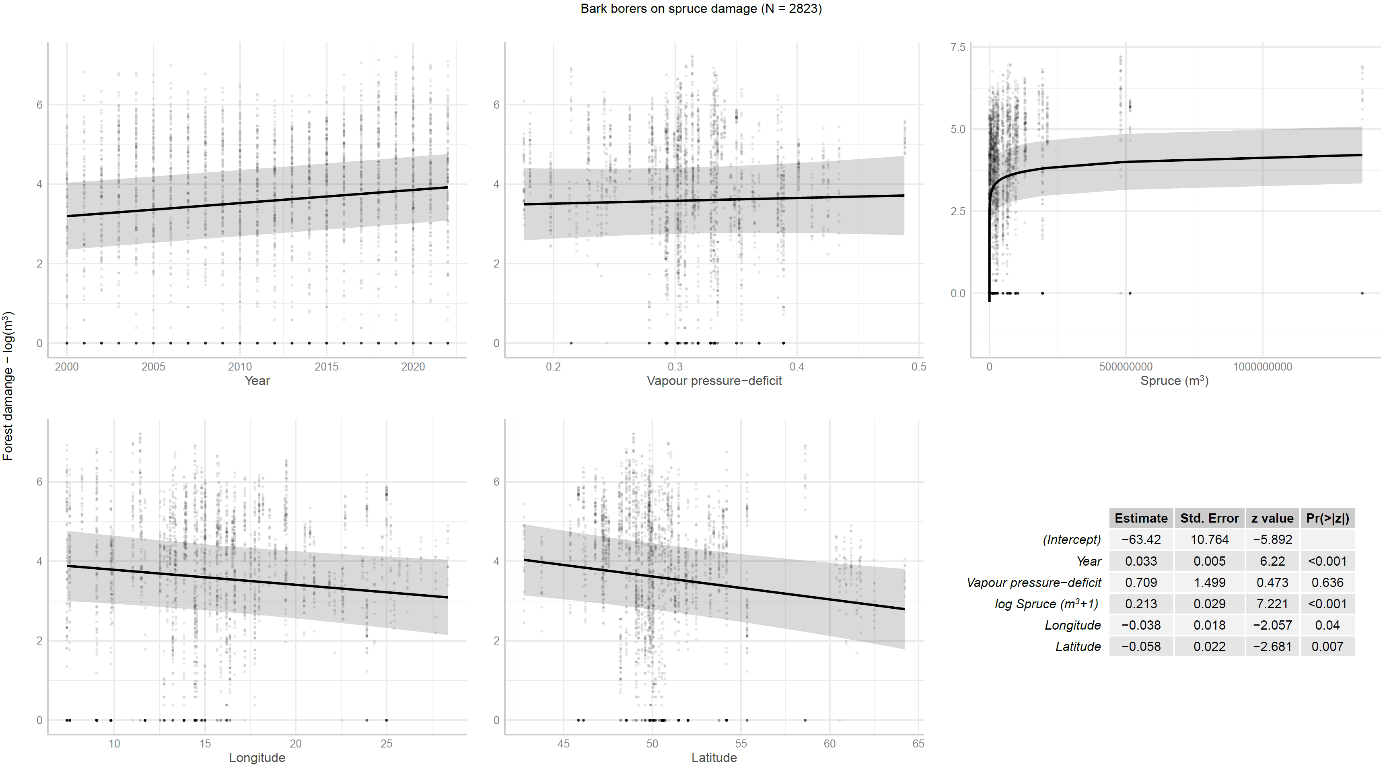


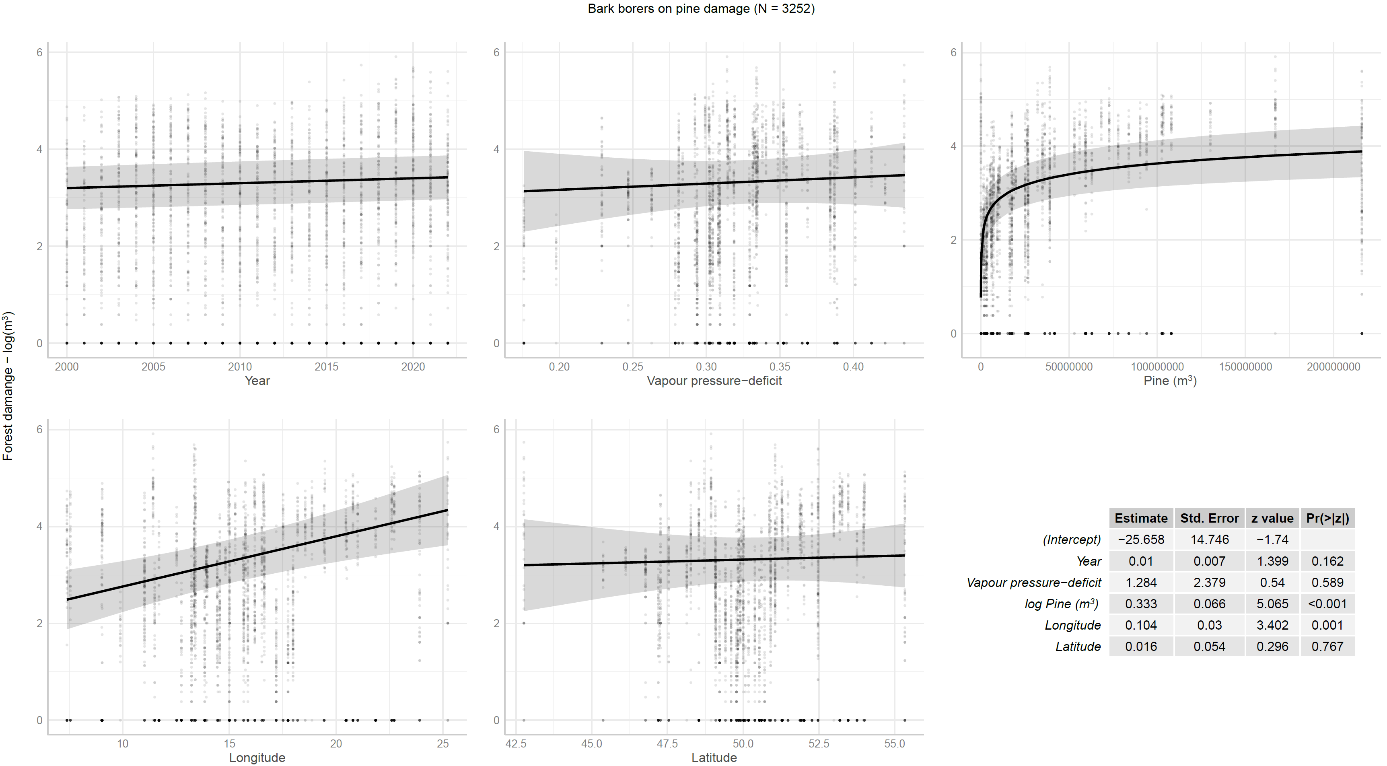


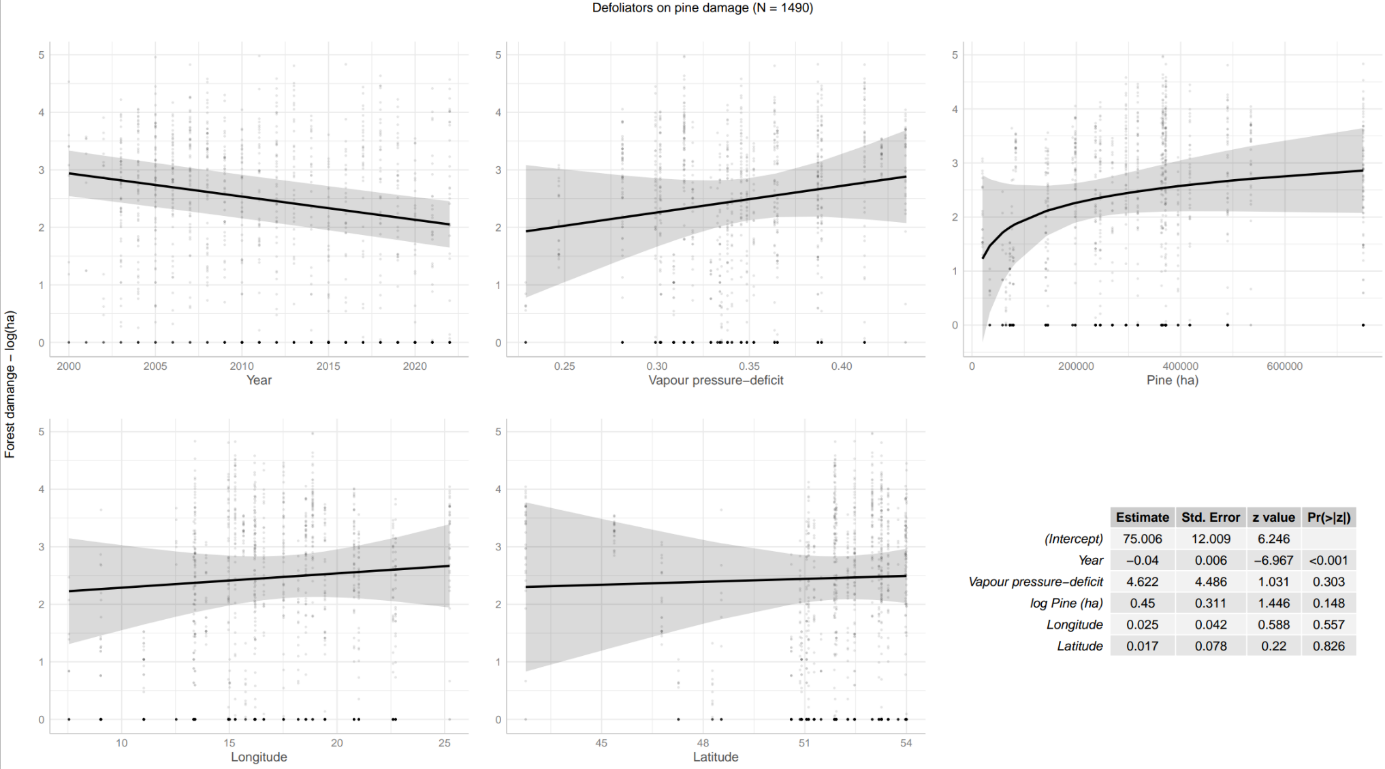


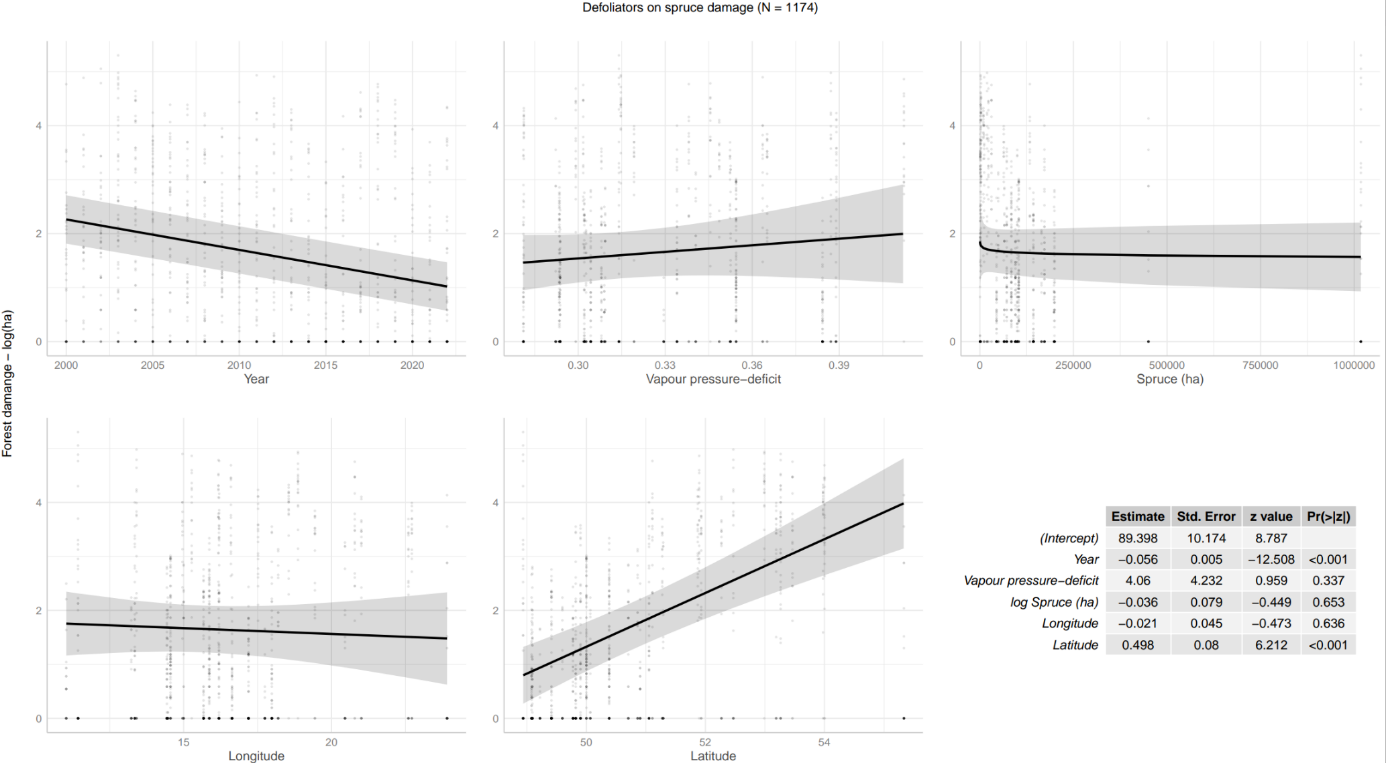


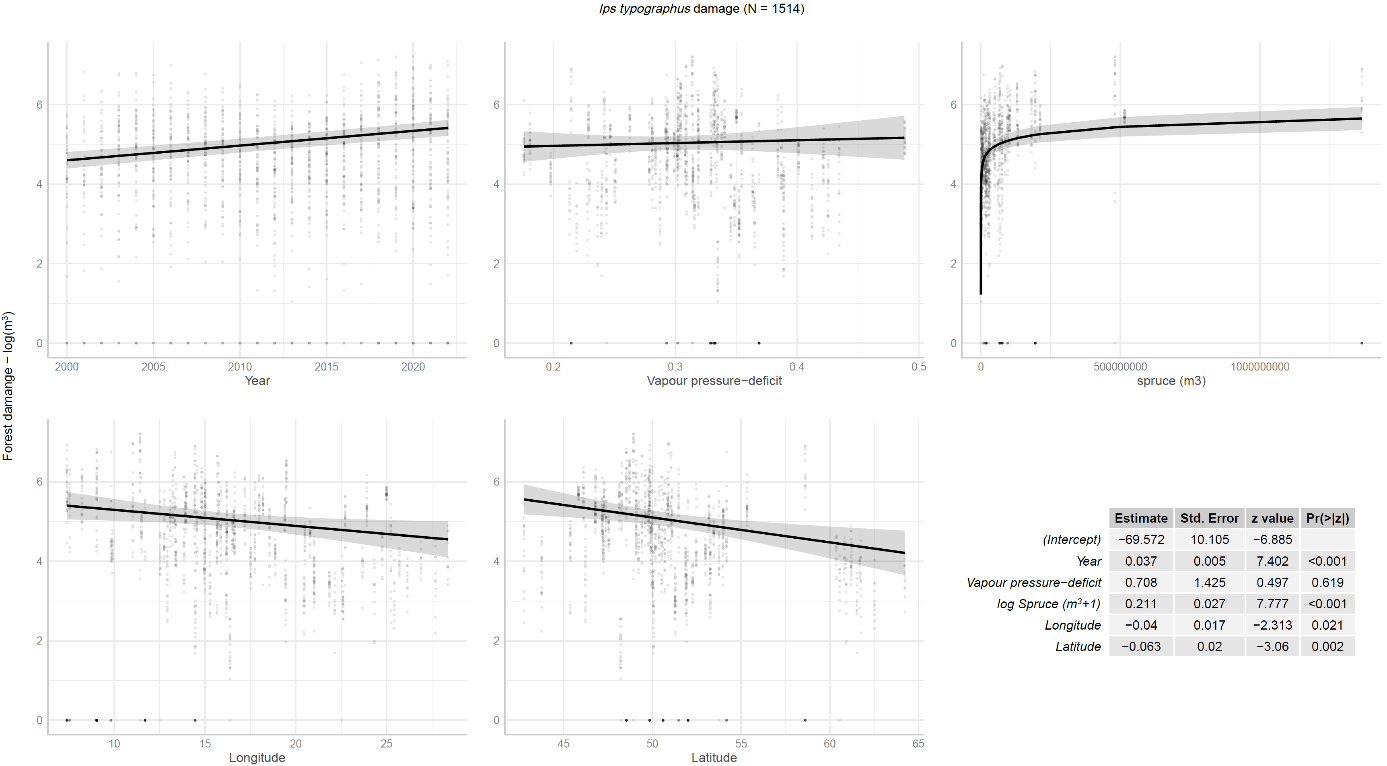


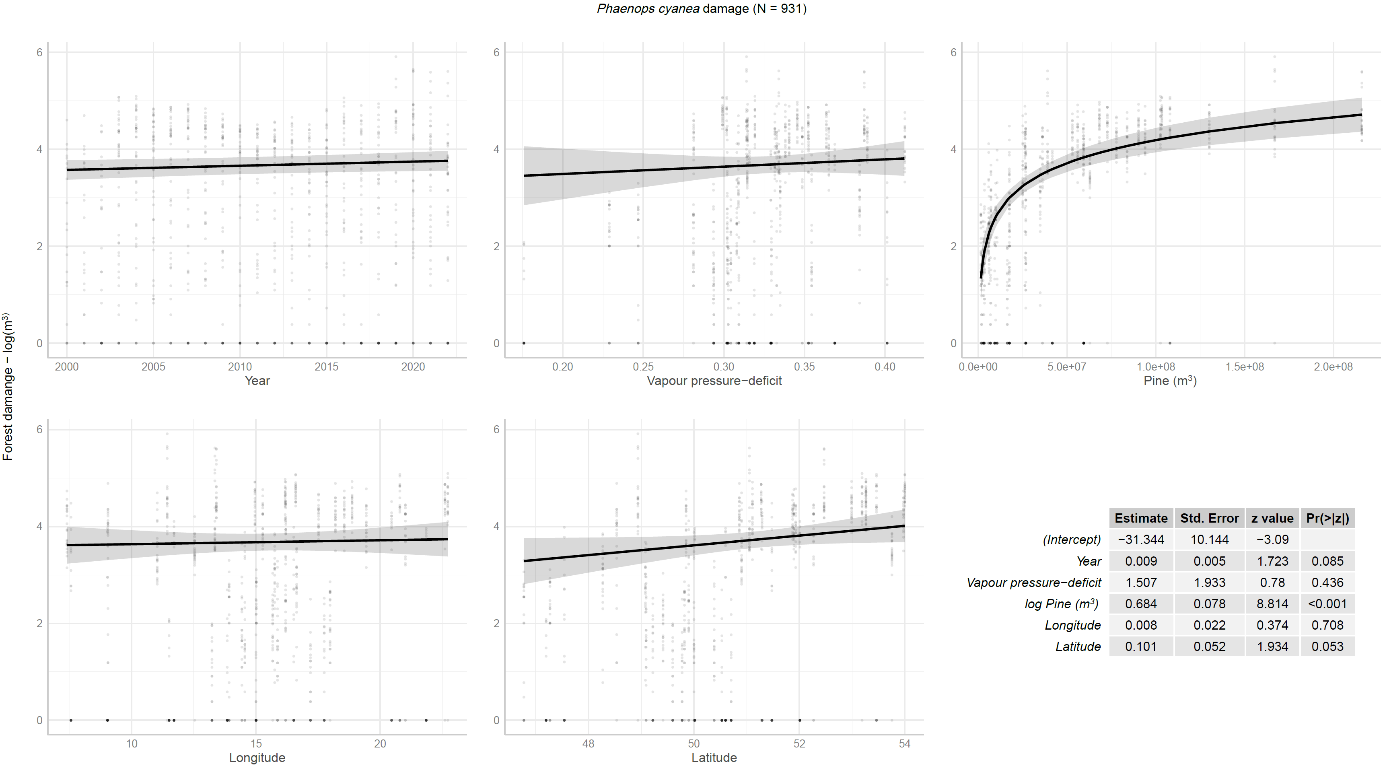


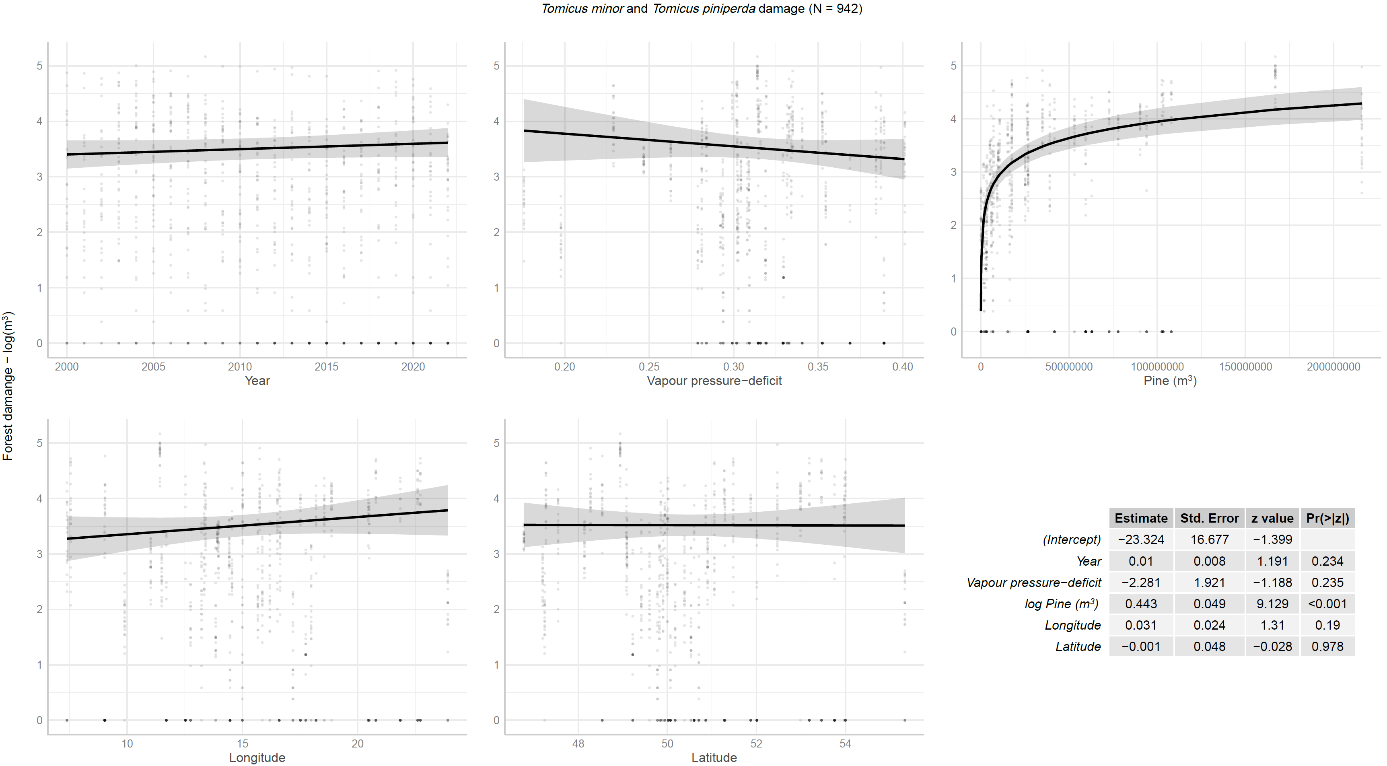


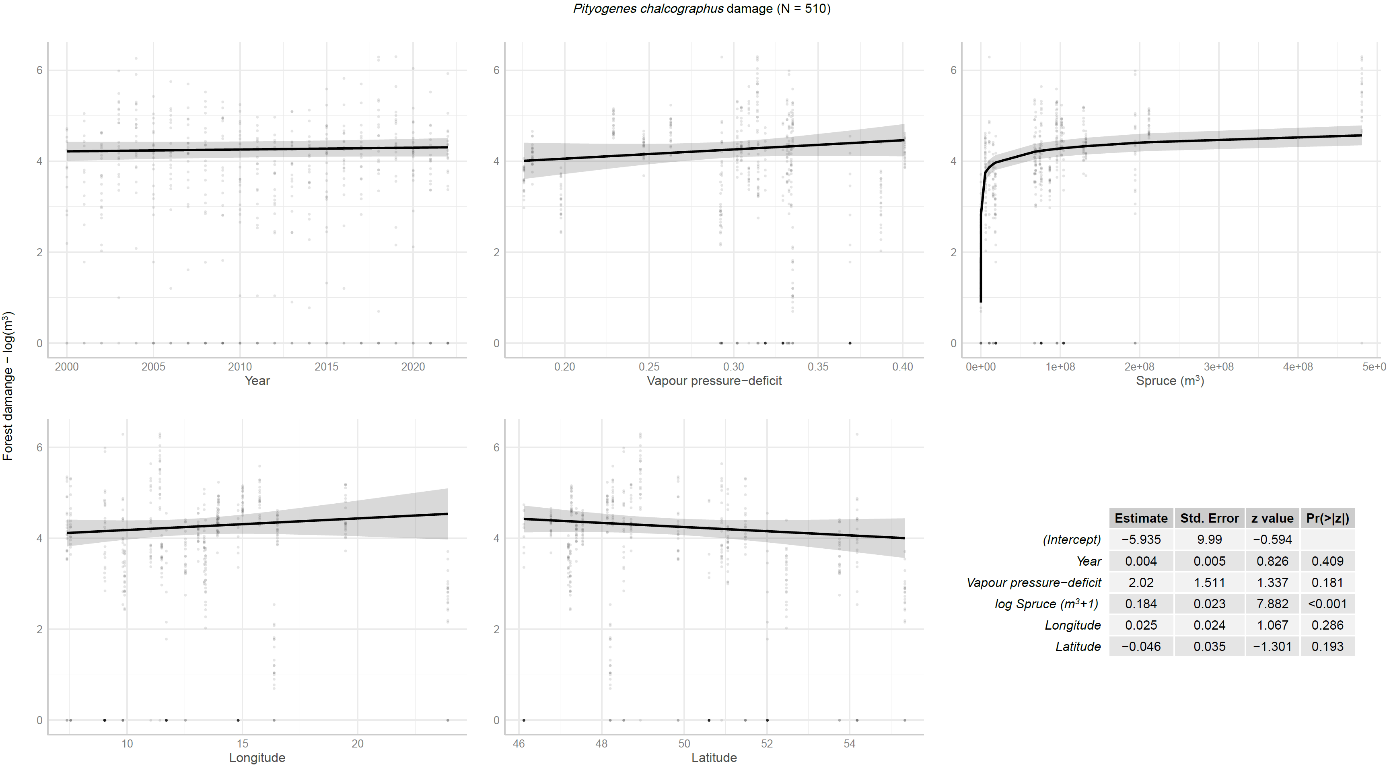


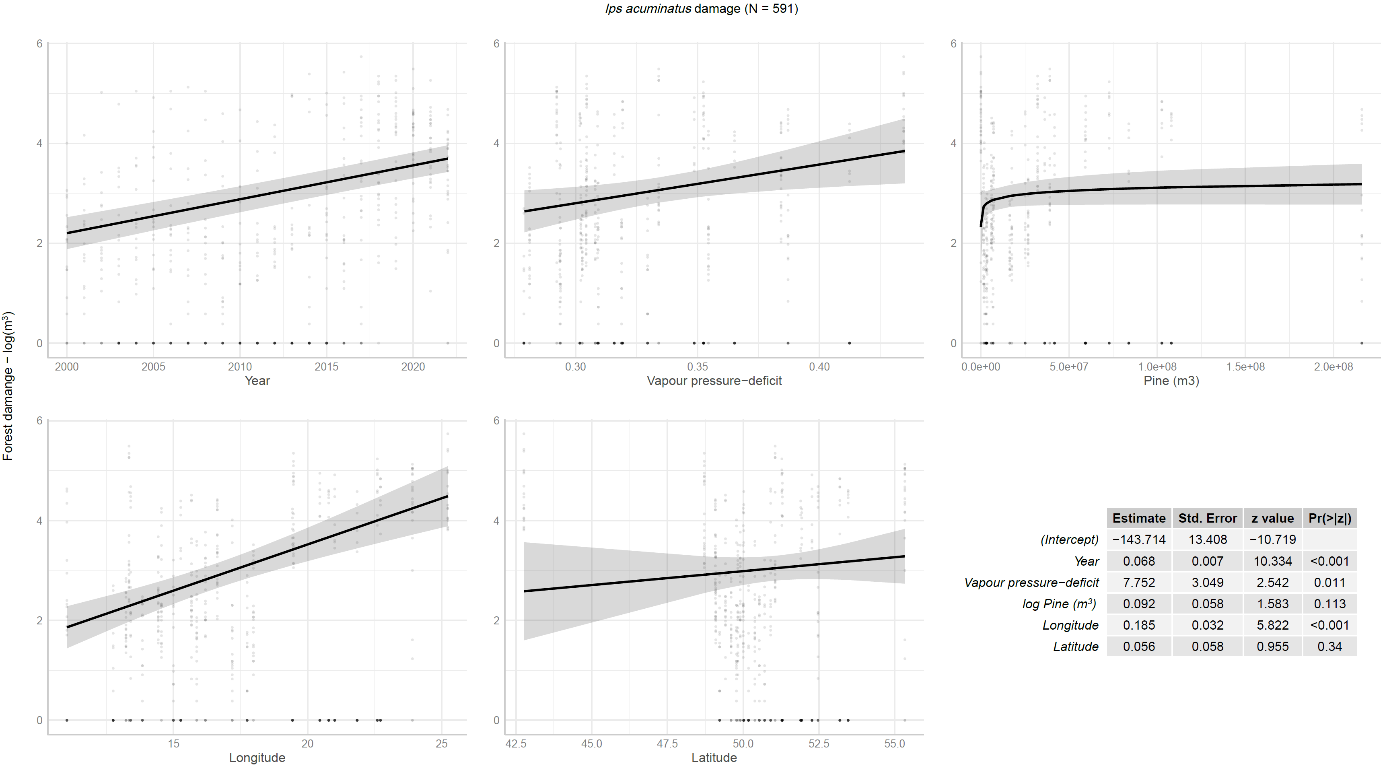


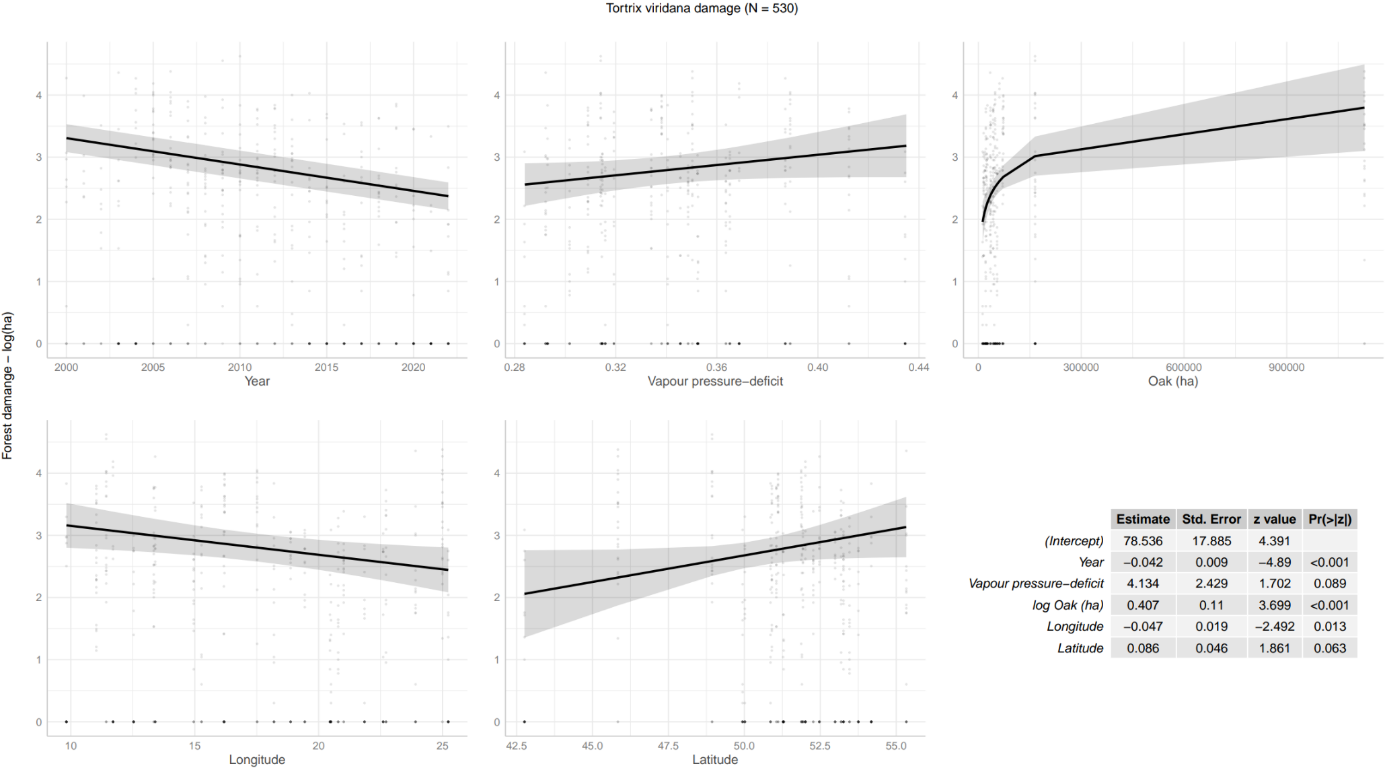


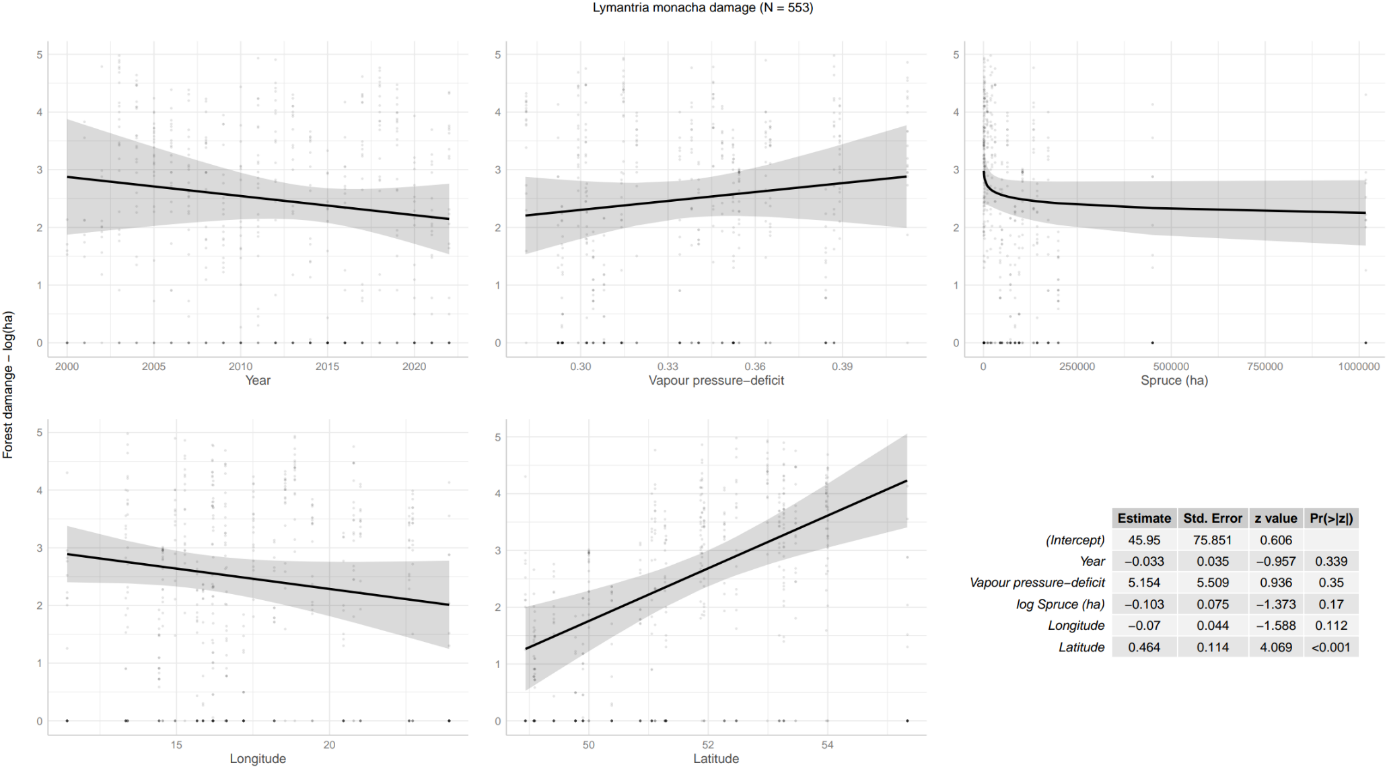


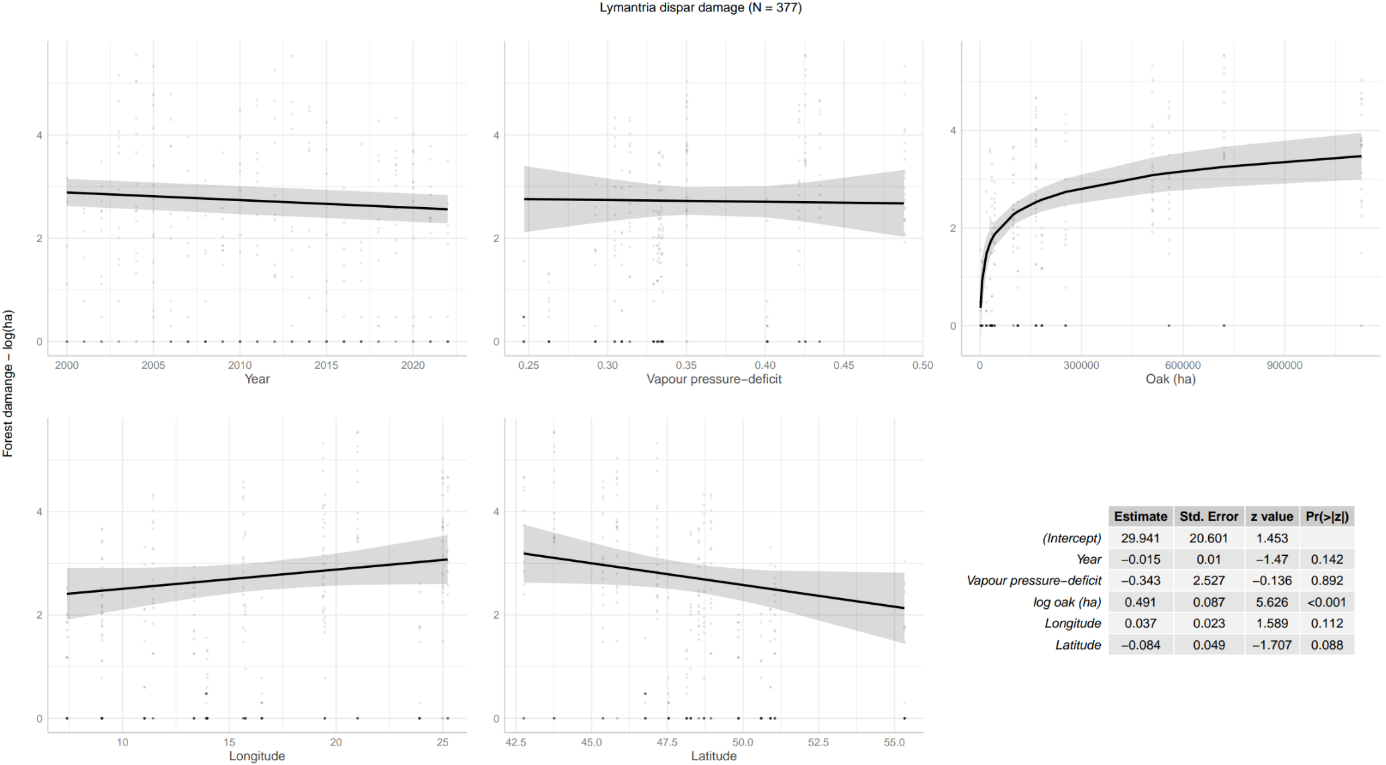


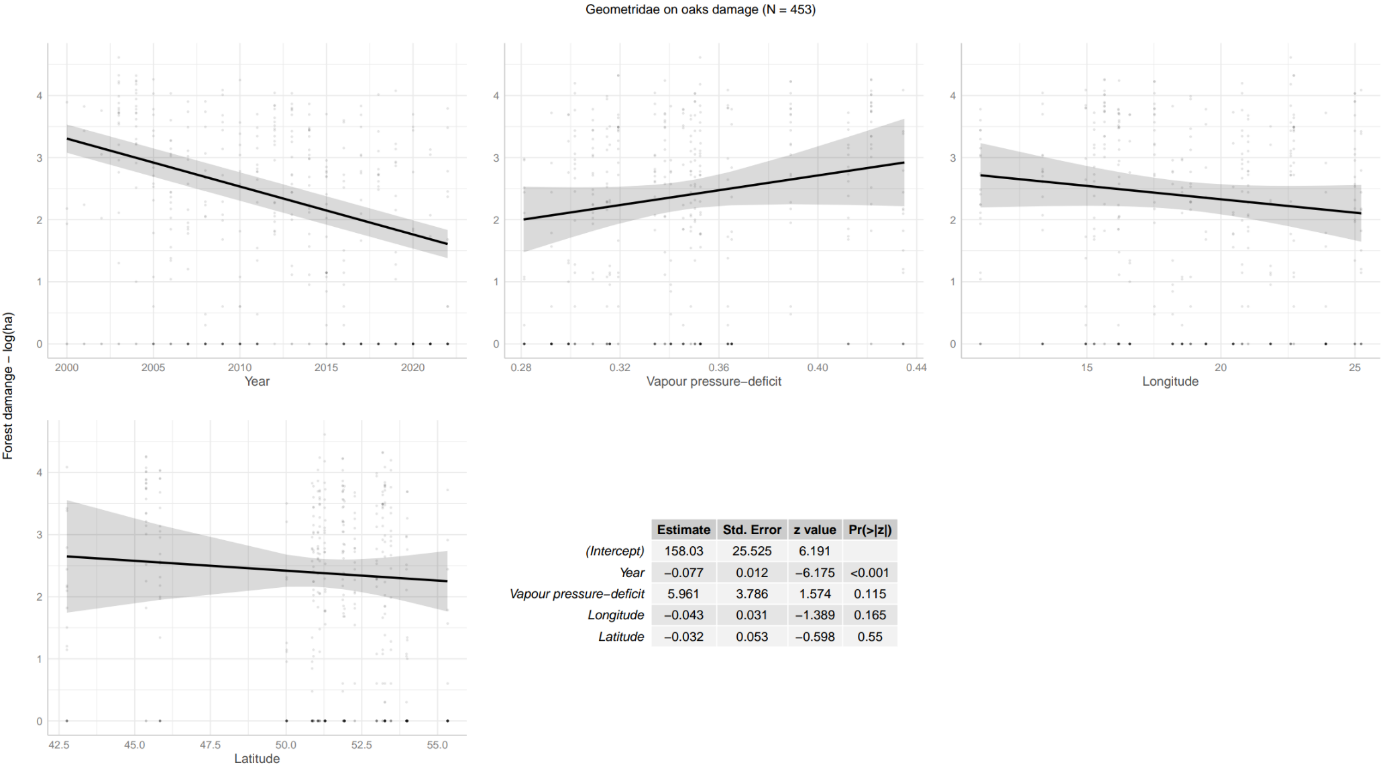

Supplement: Supplementary file 1 — Appendix S1: gcb70580‐sup‐0001‐Appendices.docx. [file GCB-31-e70580-s001.docx]
